# Identification of novel leads as potent inhibitors of HDAC3 using ligand-based pharmacophore modeling and MD simulation

**DOI:** 10.1038/s41598-022-05698-7

**Published:** 2022-02-02

**Authors:** Navanath Kumbhar, Snehal Nimal, Sagar Barale, Subodh Kamble, Rohit Bavi, Kailas Sonawane, Rajesh Gacche

**Affiliations:** 1 Department of Biotechnology, Savitribai Phule Pune University Pune, Pune, Maharashtra (MS) 411007 India; 2 Department of Microbiology, Shivaji University, Kolhapur, Maharashtra (MS) 416004 India; 3 Structural Bioinformatics Unit, Department of Biochemistry, Shivaji University, Kolhapur, Maharashtra (MS) 416004 India; 4 School of Chemical Science, Punyashlok Ahilyadevi Holkar Solapur University, Solapur, Maharashtra (MS) 413255 India

**Keywords:** Biophysics, Computational biology and bioinformatics, Drug discovery

## Abstract

In the landscape of epigenetic regulation, histone deacetylase 3 (HDAC3) has emerged as a prominent therapeutic target for the design and development of candidate drugs against various types of cancers and other human disorders. Herein, we have performed ligand-based pharmacophore modeling, virtual screening, molecular docking, and MD simulations to design potent and selective inhibitors against HDAC3. The predicted best pharmacophore model ‘Hypo 1’ showed excellent correlation (*R*^2^ = 0.994), lowest RMSD (0.373), lowest total cost value (102.519), and highest cost difference (124.08). Hypo 1 consists of four salient pharmacophore features viz. one hydrogen bond acceptor (HBA), one ring aromatic (RA), and two hydrophobic (HYP). Hypo 1 was validated by Fischer's randomization with a 95% of confidence level and the external test set of 60 compounds with a good correlation coefficient (*R*^2^ = 0.970). The virtual screening of chemical databases, drug-like properties calculations followed by molecular docking resulted in identifying 22 representative hit compounds. Performed 50 ns of MD simulations on top three hits were retained the salient π-stacking, Zn^2+^ coordination, hydrogen bonding, and hydrophobic interactions with catalytic residues from the active site pocket of HDAC3. Total binding energy calculated by MM-PBSA showed that the Hit 1 and Hit 2 formed stable complexes with HDAC3 as compared to reference TSA. Further, the PLIP analysis showed a close resemblance between the salient pharmacophore features of Hypo 1 and the presence of molecular interactions in co-crystallized FDA-approved drugs. We conclude that the screened hit compounds may act as potent inhibitors of HDAC3 and further preclinical and clinical studies may pave the way for developing them as effective therapeutic agents for the treatment of different cancers and neurodegenerative disorders.

## Introduction

Epigenetic dysregulations are the major causes of cancer which may initiate the oncogenes transcription and inactivation of tumor-suppressor genes^1^. These epigenetic alterations are closely associated with cancer progression and metastasis^2^. Amongst the variety of epigenetic modulations, the post-translational acetylation of lysine residues in the tail of histone proteins by histone acetyltransferases leads to chromatin remodeling and transcription activation^3^. In contrast, the lysine deacetylation by histone deacetylases (HDACs) is associated with a more condensed chromatin state and altered gene transcription which results in cancer progression^4,5^. Therefore, HDACs have been identified as an attractive therapeutic target for the development of novel anti-cancer agents. The vorinostat, panobinostat, belinostat and romidepsin are some of the HDAC targeting drugs currently used for treating human cancers^6,7^. HDACs are the class of zinc-dependent metalloenzymes that profoundly take part in cellular migration and invasion in many cancer subtypes. Based on their structure, functions, and evolutionary conservation, the 18 isomers of mammalian HDACs are classified into Class I (HDAC 1, 2, 3, and 8), Class II (HDAC 4, 5, 6, 7, 9, and 10), Class III (Sirtuins 1–7), and Class IV (HDAC 11)^8^.

Among Class I HDACs, the HDAC3 is aberrantly expressed in many malignancies and plays a significant role in the progression of cancer and many other human diseases^9,10^. High expression of HDAC3 promotes cell proliferation, differentiation, migrations, stemness, and chemoresistance in human colorectal cancer^11,12^. The elevated expression of HDAC3 has been attributed to the downregulation of a series of tumor suppressor micro-RNAs including miR-296-3p, miR-451, and miR-495-3p in colorectal, melanoma, and prostate cancer, respectively^13–15^. Moreover, the high expression of HDAC3 is linked with tumorigenesis and breast cancer brain metastases^14^. Therefore, HDAC3 acts as an independent prognostic biomarker for brain metastasis-free survival in breast cancer patients. HDAC3 is an epigenetic regulator of a variety of cell signaling pathways, also the knockdown of HDAC3 promotes the G0/G1 cell cycle arrest which subsequently induces cell apoptosis in acute myeloid leukemia, prostate, melanoma, and colorectal cancer^13–17^. A plethora of literature describes the pathophysiological role of HDAC3 in a variety of human cancers, neurodegenerative disorders, diabetes, inflammatory and cardiovascular diseases^18–25^.

Therefore, considering the important role of HDAC3 in variety of cancer subtypes and various other human ailments, many studies have been performed for design and development of selective HDAC3 inhibitors^10,26–31^. For example, HDAC3 inhibition by selective inhibitor RGFP966 has restored the expression of the BRM tumor suppressor gene in renal cell carcinoma and inhibited the growth of glioma stem cells^19,32^. In addition, the RGFP966 also decreased the expression of Aβ protein and improved the learning, memory ability in Alzheimer's patients and also protected the ischemic brain damage by down-regulating the absent in melanoma 2 inflammasome in microglia^22,33^. Moreover, HDAC3 inhibitors were effectively tested on various cancer subtypes, neurodegenerative disorders, cardiac diseases, atherosclerosis, human immunodeficiency virus, and inflammatory diseases^5–7,34–37^. However, besides a better therapeutic profile, the present HDAC3 inhibitors have demonstrated disappointing clinical outcomes due to serious side effects and off-target toxicity in treated patients. To circumvent these issues, many computational strategies have been employed for the design and development of novel, safe and selective inhibitors of HDAC3^38–40^.

In the mainstream of drug discovery, the three dimensional quantitative structure relationship (3D-QSAR) and ligand/structure-based pharmacophore modeling approaches have established a reputation towards designing novel, potent and selective inhibitors against HDACs^38–42^. For example, the QSAR modeling was performed using k-nearest neighbor (kNN) and support vector machines (SVM) to design the structurally novel bioactive compound against HDAC1^43,44^. Pharmacophore-based virtual screening and MD simulations were used to design the novel hydroxamic acids and non-hydroxamate derivatives as potential inhibitors of HDAC2, HDAC4, and HDAC6^45–48^. Series of similar in silico settings have been undertaken to identify potential inhibitors of HDAC8 using docking-enabled pharmacophore, comparative molecular field analysis CoMFA) and comparative molecular similarity indices analysis (CoMSIA) techniques^49,50^.

A vast body of literature has accumulated in the recent past linking the role of 3D-QSAR and pharmacophore modeling in the discovery of class I and II HDAC inhibitors (1, 2, 4, 6, and 8). Few computational studies have also been performed to understand the structural and physicochemical properties of benzamide-based HDAC3 inhibitors using the 3D-QSAR (CoMFA/CoMSIA) approach and HDAC3i-Finder online tool^51,52^. The virtual screening and in-vitro studies were performed to discover the potent and selective inhibitors against HDAC3^53^. Considering the important role of HDAC3 in pathophysiological conditions of many human diseases, the knowledge of pharmacophore features is required for the development of selective HDAC3 inhibitors remains elusive. Therefore, in the present investigation, we performed ligand-based pharmacophore modeling, virtual screening, and MD simulation studies, Molecular mechanics-Poisson-Boltzmann solvent accessible surface area (MM-PBSA), and principal component analysis (PCA) to identify potent HDAC3 inhibitors with different structural scaffolds.

## Results

### Pharmacophore model generation

**Figure 1:**
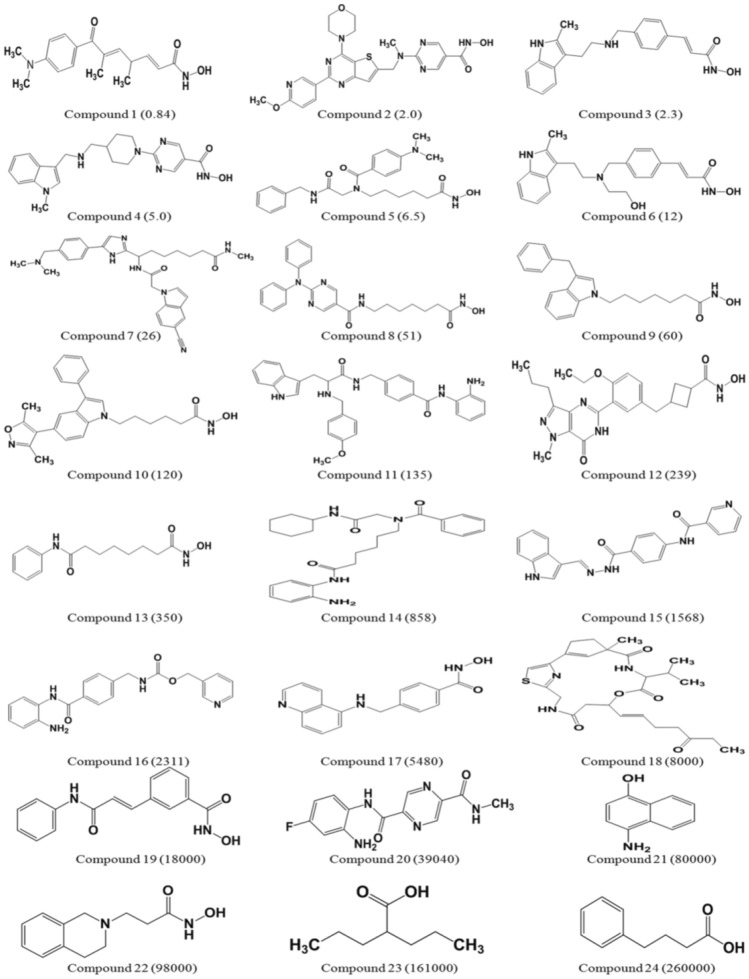
A representation of the 24 chemically diverse training set compounds used for pharmacophore generation. The experimental IC_50_ values (nmol/L) are shown in parentheses for each compound. The figure is drawn using ChemDraw Ultra v12.0.2.

A dataset of 24 chemically diverse HDAC3 inhibitors with IC_50_ values spanning over the four orders of magnitude (0.84 nmol/L to 260,000 nmol/L) was selected for the generation of the 3D-QSAR pharmacophore model using the *HypoGen* algorithm (Fig. 1).

**Figure 2:**
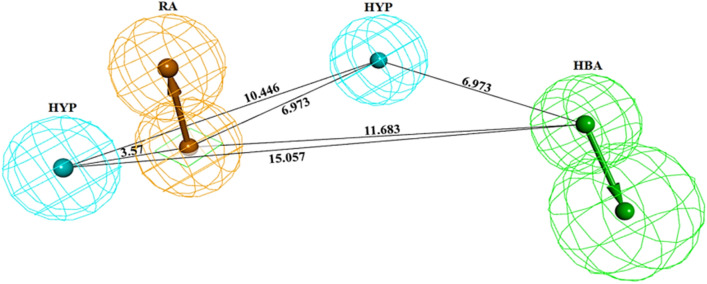
The best pharmacophore model ‘Hypo 1’, with distance constraints. Hypo 1 contains one hydrogen bond acceptor (HBA: green), one ring aromatic (RA: orange), and two hydrophobic regions (HYP: cyan).The figure is drawn using DS v3.5.

A total of 10 hypotheses were generated that correlated the experimental and predicted HDAC3 inhibitory activity values of the training set inhibitors. The best pharmacophore hypothesis (Hypo 1) was selected based on the statistical parameters such as lowest total cost values, highest cost difference, high correlation coefficient, and smallest RMSD values. The cost difference is the difference between the null cost and the total cost of each generated hypothesis. The null cost, fixed cost, and configuration costs for the top 10 scored hypotheses were found to be 217.69, 91.93, and 16.80, respectively. A cost difference greater than 60 indicates a high correlation between the experimental and estimated activities whereas, a cost difference less than 40 represents the predictive ability of the model is below 50%. The configuration cost value (16.80) of all ten generated hypotheses indicating the generated hypotheses are reasonably of good quality (Table 1). The Hypo 1 consisted of four salient chemical features including one HBA, one RA, and two HYP (Fig. 2 and Table 1). The 3D-spatial arrangement of chemical features and distance constraints between them are depicted in Fig. 2. Hypo 1 showed the lowest cost value (102.519), the highest cost difference (124.08), the maximum correlation coefficient (*R*^2^ = 0.994), and the smallest RMSD (0.373) value.

**Table 1:** Statistical results of ten pharmacophore hypotheses generated by HypoGen.

Hypo no	Total cost	Cost difference	RMSD^b^	Correlation (*R*^2^)	Max fit	Features^c^
Hypo 1	102.519	124.08	0.373	0.994	10.524	HBA, HYP, HYP, RA
Hypo 2	105.239	121.36	0.516	0.989	11.142	HBA, HYP, HYP, RA
Hypo 3	109.977	116.63	0.852	0.970	10.749	HBA, HYP, HYP, RA
Hypo 4	110.475	116.13	0.837	0.971	11.148	HBA, HYP, HYP, RA
Hypo 5	111.065	115.54	0.921	0.965	10.541	HBA, HYP, HYP, RA
Hypo 6	111.336	115.27	0.970	0.960	9.998	HBA, HYP, HYP, RA
Hypo 7	112.760	113.84	0.975	0.960	10.793	HBA, HYP, HYP, RA
Hypo 8	112.979	113.62	0.905	0.967	11.620	HBA, HYP, HYP, RA
Hypo 9	113.090	113.51	0.998	0.958	10.678	HBA, HYP, HYP, RA
Hypo 10	115.965	110.64	1.199	0.938	8.376	HBD, HYP, HYP, RA

^a^Cost difference, the difference between the null cost and the total cost. The null cost of ten scored hypotheses is 226.602, the fixed cost value is 98.657, and the configuration cost is 16.80. All costs are represented in bit units.

^b^RMSD: deviation of the log (estimated activities) from the log (experimental activities) normalized by the log (Uncertainties).

^c^HBA: Hydrogen Bond Acceptor, HYP: Hydrophobic, RA: Ring Aromatic.

**Figure 3:**
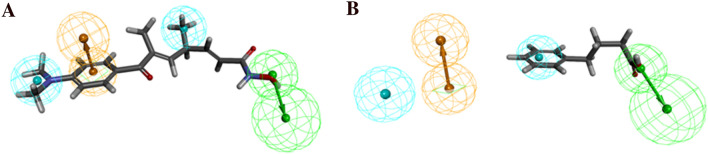
Alignment of Hypo 1 to training set compounds; (**A**) Most active compound 1 (IC_50_ = 0.84 nmol/L) and (**B**) Least active compound 24 (IC_50_ = 260,000 nmol/L). The most active compound mapped to all four features in Hypo 1, whereas the least active compound missed the RA and HYP features. The figure is drawn using DS v3.5.

The cost difference (124.08) greater than 60 indicates that the model has good predictive accuracy, represented the 90% of the true correlation. A total cost (102.519) of Hypo 1 was almost close to the fixed cost (98.65). This confirms that the Hypo 1 was not generated by chance. The high correlation coefficient represented a good predictive ability of the Hypo 1 whereas, the lower RMSD value indicated a small deviation in the experimental and estimated activity values. Therefore, Hypo 1 was used to predict the HDAC3 inhibitory activity amongst the 24 training set compounds that conversely determined the predictive ability of Hypo 1. The most active (IC_50_ = 0.84 nmol/L) and least active (IC_50_ = 260,000 nmol/L) compounds from training set were aligned to Hypo 1 (Fig. 3).

The most active compound has mapped all pharmacophore features from Hypo 1 whereas, the least active compound has mapped only two (HBA and HYP) features and missed RA and HYP features. Hypo 1 efficiently discriminated the active, moderately active, and inactive compounds from the training set with a high degree of accuracy (Table 2). Hypo 1 has predicted the inhibitory activity values of all compounds in the same order of magnitude, except for one active compound which was underestimated as moderately active (Table 2). The ratio between the experimental and estimated activity values of training set compounds has been termed the error value. The small error value represents the high correlation between experimental and estimated activities and *vice-versa*. Hypo 1 was validated using Fischer's randomization method and the external test dataset.

**Table 2:** The experimental and predicted activity of training set compounds based on Hypo 1.

Compd. no	Fit value	Experimental IC_50_ (nmol/L)	Predicted IC_50_ (nmol/L)	Error^a^	Experimental scale^b^	Predicted scale^b^
1	10.140	0.84	1.3	1.60	+++	+++
2	9.820	2.0	2.8	1.40	+++	+++
3	9.580	2.3	4.7	2.10	+++	+++
4	9.610	5.0	4.5	− 1.10	+++	+++
5	9.470	6.5	6.1	− 1.10	+++	+++
6	9.230	12	11	− 1.10	+++	+++
7	8.740	26	33	1.30	+++	+++
8	8.840	51	26	− 2.0	+++	+++
9	8.240	60	110	1.80	+++	++
10	7.980	120	190	1.60	++	++
11	8.160	135	120	− 1.10	++	++
12	7.700	239	360	1.50	++	++
13	7.700	350	360	1.0	++	++
14	7.440	858	660	− 1.30	++	++
15	7.200	1568	1100	− 1.40	++	++
16	7.100	2311	1400	− 1.60	++	++
17	6.390	5480	7500	1.40	++	++
18	6.670	8000	3900	− 2.10	++	++
19	6.080	18,000	15,000	− 1.20	+	+
20	5.720	39,040	34,000	− 1.10	+	+
21	5.170	80,000	120,000	1.50	+	+
22	5.260	98,000	99,000	1.0	+	+
23	5.020	161,000	170,000	1.10	+	+
24	5.220	260,000	110,000	− 2.40	+	+

^a^Values in the error column represent the ratio of the estimated activity (Pred IC_50_) to the experimental activity (Exp IC_50_) or its negative inverse if the ratio is < 1.

^b^Activity scale: IC_50_ < 100 nmol/L = +++ (active), 100 nmol/L ≤ IC_50_ < 10,000 nmol/L =  ++ (moderate active), IC_50_ ≥ 10,000 nmol/L =  + (inactive).

### Hypothesis validation

#### Fischer randomization

**Figure 4:**
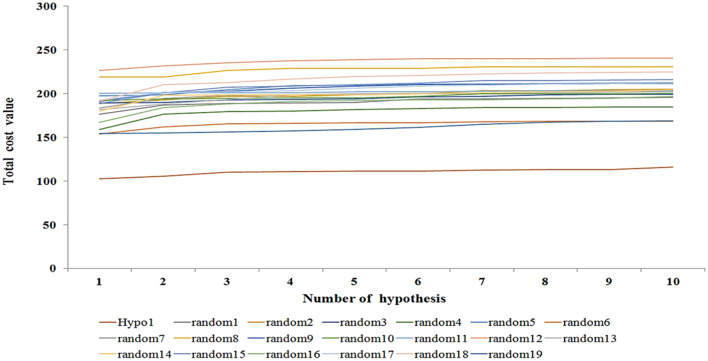
A graphical representation of the total cost values of Hypo 1 and each of ten hypotheses generated from 19 random spreadsheets during Fischer’s randomization run. A confidence level of 95% was used. The figure is drawn using Microsoft Excel 2013 v15.0.

Fischer’s randomization method was employed to assess the statistical significance of each generated pharmacophore model to ensure that the chosen pharmacophore model was significantly better than the nine others obtained from the combinations of pharmacophore features (Fig. 4).

In the Fischer randomization run, a set of 19 random spreadsheets were generated from the random combinations of the pharmacophore features at a 95% confidence level and compared to the ‘Hypo 1’. The fixed cost value of these 19 random models along with the best pharmacophore model is given in Fig. 4. The total cost value of Hypo 1 was the least as compared to other hypotheses in the generated random spreadsheets. Thus, Fischer’s randomization results indicated that Hypo 1 is far more superior compared to all other random hypotheses and not generated by chance.

#### Test set validation

**Figure 5:**
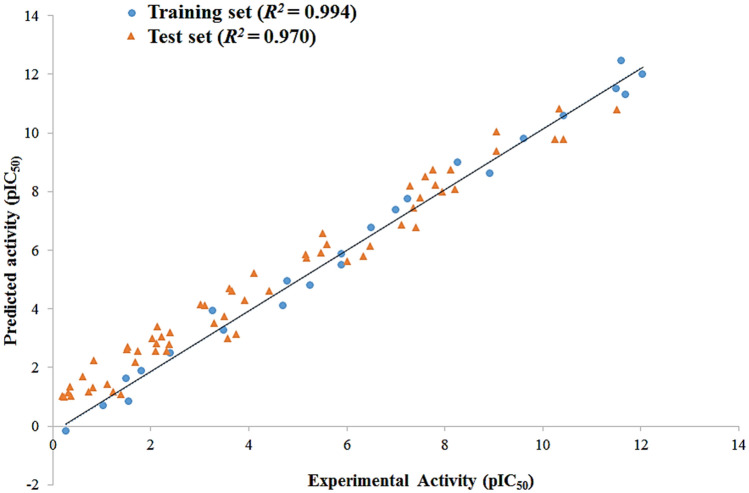
Correlations between the experimental activities and the predicted activities using Hypo 1 with the test set and training set compounds. The figure is drawn using Microsoft Excel 2013 v15.0.

The predictive ability of Hypo 1 was further assessed by performing external test set validation to predict and classify the compounds according to their correct activity range (Table 3, S1 Table 1). The dataset of 60 structurally diverse compounds having HDAC3 inhibitory activity was categorized as active, moderately active, and inactive based on their activity scale, respectively (Table 3 and S1 Table). The estimated activity values of test set compounds and their predicted activity scale along with the error between them are given in Table 3. Hypo 1 was able to classify all test set compounds according to their activity ranges except for two moderately active and one inactive compound, which were overestimated as active and moderately active, respectively. The simple regression between the experimental and estimated activity values of the test set compounds showed a strong correlation coefficient value of 0.970, which is evident by the low error values (Fig. 5). Thus, these results confirm that Hypo 1 has an excellent predictive capability to discriminate the active compounds from the moderately active and inactive compounds from both training and test datasets (Table 3).

**Table 3:** Evaluation of predicted and experimental activity (IC_50_) values of test set compounds using Hypo 1.

Compd. no	Fit value	Experimental IC_50_ (nmol/L)	Predicted IC_50_ (nmol/L)	Error^a^	Experimental scale^b^	Predicted scale^b^
1	10.15	2.7	1.25	− 2.16	+++	+++
2	10.09	2.8	1.44	− 1.94	+++	+++
3	10.17	2.8	1.20	− 2.34	+++	+++
4	9.65	3.0	3.98	1.32	+++	+++
5	10.12	3.1	1.36	− 2.27	+++	+++
6	8.74	3.2	3.39	1.05	+++	+++
7	9.93	3.2	2.08	− 1.53	+++	+++
8	9.90	3.8	2.26	− 1.6	+++	+++
9	10.10	3.9	1.40	− 2.78	+++	+++
10	9.77	4.2	3.00	− 1.4	+++	+++
11	9.99	5.5	1.83	− 3	+++	+++
12	9.52	9.0	5.40	− 1.66	+++	+++
13	9.89	9.3	2.29	− 4.06	+++	+++
14	9.25	13	10.11	− 1.28	+++	+++
15	9.50	13	5.70	− 2.2	+++	+++
16	9.34	13	8.14	− 1.59	+++	+++
17	9.60	13.9	4.48	− 3.09	+++	+++
18	9.59	15	4.60	− 3.2	+++	+++
19	9.22	16.3	10.66	− 1.52	+++	+++
20	9.33	17	8.31	− 2.04	+++	+++
21	9.38	20	7.52	− 2.65	+++	+++
22	8.71	20	35.24	1.76	+++	+++
23	9.29	21	9.25	− 2.27	+++	+++
24	8.63	23	42.14	1.83	+++	+++
25	9.21	24.7	10.96	− 2.25	+++	+++
26	9.33	30	8.39	− 3.57	+++	+++
27	8.83	34	26.72	− 1.27	+++	+++
28	8.74	42.5	32.74	− 1.29	+++	+++
29	8.91	62	22.03	− 2.81	+++	+++
30	8.94	63	20.34	− 3.09	+++	+++
31	8.55	74	50.41	− 1.46	+++	+++
32	8.33	100	82.74	− 1.2	+++	+++
33	8.66	100	38.78	− 2.57	+++	+++
34	8.69	110	36.58	− 3	++	+++
35	8.47	187	60.57	− 3.08	++	+++
36	7.64	276	408.24	1.47	++	++
37	8.01	310	176.33	− 1.75	++	++
38	7.50	330	559.72	1.69	++	++
39	8.01	354	174.64	− 2.02	++	++
40	7.88	374	238.12	− 1.57	++	++
41	7.44	464	651.11	1.37	++	++
42	7.83	495	267.27	− 1.85	++	++
43	7.86	719	247.03	− 2.91	++	++
44	7.03	887	1658.49	1.86	++	++
45	7.17	967	1219.99	1.26	++	++
46	7.06	1700	1575.90	− 1.07	++	++
47	7.00	2400	1809.01	− 1.32	++	++
48	6.81	3000	2795.99	− 1.07	++	++
49	6.69	3200	3663.62	1.14	++	++
50	7.09	3600	1451.96	− 2.47	++	++
51	6.86	3700	2459.29	− 1.5	++	++
52	6.95	4989	1992.83	− 2.5	++	++
53	6.89	6200	2326.25	− 2.66	++	++
54	6.73	6330	3359.50	− 1.88	++	++
55	6.32	12,000	8480.08	− 1.41	+	++
56	5.81	18,000	27,941.10	1.55	+	+
57	5.73	18,000	33,447.30	1.85	+	+
58	6.32	23,000	8475.24	− 2.71	+	+
59	5.26	48,200	98,932.50	2.05	+	+
60	5.77	50,200	30,552.50	− 1.64	+	+

### Virtual screening and drug-likeness filtration

**Figure 6:**
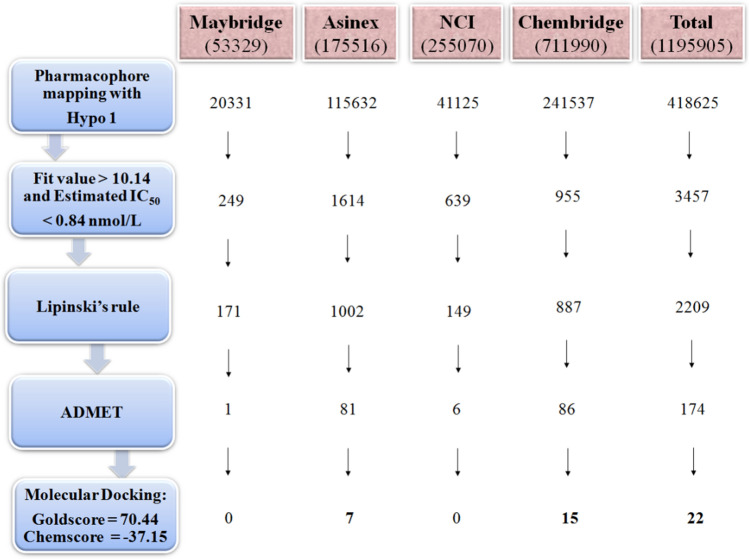
The summary of the virtual screening using Hypo 1. The figure is drawn using Microsoft PowerPoint 2013 v15.0.

The chemical features of the best pharmacophore model play a critical role in the mapping and screening of novel hit compounds from the chemical databases containing structurally different scaffolds and diversified functional groups. The novel HDAC3 inhibitors were discovered by searching the Maybridge (53329), Asinex (175516), NCI (255070), and Chembridge (711990) databases using Hypo 1 as a 3D structural query. A total of 418625 hit compounds were retrieved which mapped all chemical features of Hypo 1 (Fig. 6). Using the estimated IC_50_ value < 0.84 nmol/L and maximum fit value > 10.14 (the highest fit value for the active compound from the training set) as a cut-off, the retrieved hit compounds were sorted to 3457. Further, the obtained hits were tested for drug-likeness properties using Lipinski’s Rule of Five and ADMET property calculations. The 2209 hits successfully satisfied Lipinski's rule, and calculated properties were helpful to investigate the good oral bioavailability of drugs. The ADMET calculations were performed to assess the pharmacokinetic properties of hit compounds in the human body.

Drug properties including the BBB penetration, solubility, hepatotoxicity, human intestinal absorption, CYP450 2D6 inhibition, and plasma protein binding were calculated for 2209 hit compounds. The 174 hit compounds were selected based on the drug-like properties that fulfilled the values of 3, 3, and 0 for the BBB, solubility, and absorption, respectively. The molecular docking study was performed to check the binding affinity and relative orientation of 174 hit compounds in the catalytic pocket of HDAC3.

### Molecular docking of hit compounds with HDAC3

The molecular docking studies of 174 screened hit compounds and training set compounds were performed with HDAC3 using the GOLD software. Only one crystal structure of HDAC3 (4A69.PDB) is available with acetate ion present in the active site pocket that coordinated with zinc (Zn^2+^) ions^54^. The HDAC3 has sequence and structural similarities to HDAC8 and possesses conserved catalytic active site residues^55^. The crystal structure of HDAC8 (1T64.PDB and 3EW8.PDB) contains TSA and 4-(dimethylamino)-*N*-[7-(hydroxyamino)-7-oxoheptyl] benzamide (B3N) inhibitors^56^. The exact superimposition of HDAC8 crystal structures on HDAC3 showed structural similarities and the co-crystallized inhibitors were likely coordinated with Zn^2+^ and formed molecular interactions similar to acetate ion (Figure S1). The most active compound from the training set was a TSA. Therefore, we selected the TSA-HDAC8 active site XYZ coordinates for molecular docking studies of screened hit compounds and training set compounds with HDAC3. The GOLD fitness score of the most active compound from the training set was selected as the cutoff for the selection of hit compounds. Goldscore (70.49) of the TSA was used as a cut-off for the screening of compounds, and a total of 22 compounds were selected. Further, the Chemscore was used as the rescoring function during docking studies and estimates the total free energy change that occurs upon ligand binding to the active site pocket of HDAC3. The hit compounds having lower Chemscore (− 37.15) values were selected for further analysis. The docking complexes of top scored hit compounds were analyzed for binding pattern and intermolecular interactions (Table 4).

**Table 4:** Comparison of gold fitness score, chemscore and average binding energy of docking complexes of reference inhibitor (TSA)/Hit 1/Hit 2/Hit 3 with HDAC3.

Complex name	Database	Gold fitness score	Chemscore
HDAC3 + Hit 1	Chembridge	80.67	− 38.55
HDAC3 + Hit 2	Asinex	76.01	− 42.93
HDAC3 + Hit 3	Chembridge	71.00	− 43.86
HDAC3 + TSA	Reference	70.49	− 37.15

**Figure 7:**
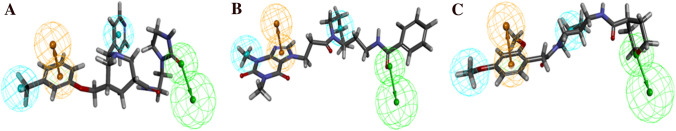
Final hit compounds mapped to the best pharmacophore model, Hypo 1; (**A**) Hit 1, (**B**) Hit 2 and (**C**) Hit 3. The HBA, RA, and HYP features are displayed in green, orange, and cyan, respectively. Hit compounds are represented as stick models. The figure is drawn using DS v3.5.

The selected three top-scored hit compounds mapped all chemical features from Hypo 1 (Fig. 7). The HDAC3 is a Zn^2+^-dependent enzyme, therefore the hit compounds coordinated with Zn^2+^ and interact with His and Tyr residues from the active site pocket HDAC3 were selected for the MD simulations studies.

### Molecular dynamics simulation study of hit compounds with HDAC3

**Figure 8:**
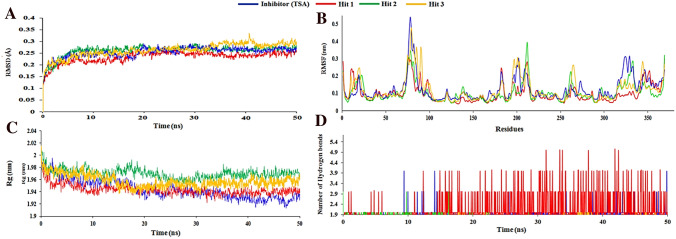
RMSD plots for checking the overall stability of the systems during 50 ns MD simulations; (**A**) RMSD profile of HDAC3 in presence of hit compounds, (**B**) RMSF of simulated complexes of HDAC3 with hit compounds, (**C**) Radius of gyration of HDAC3 with bound hit compounds and (**D**) Time dependent hydrogen bonds between HDAC3 and hit compounds. The figure is drawn using Microsoft Excel 2013 v15.

Molecular dynamics simulations of 50 ns for each hit compound complexed with HDAC3 were performed to investigate the effect of explicit water solvent on the stability of reference inhibitor (TSA) and hit compounds in the catalytic pocket of HDAC3. The simulations were analyzed to assess the binding affinity of the final hit compounds towards HDAC3. The conformational stability of HDAC3 and hit compounds were investigated by calculating the root mean square deviation (RMSD) and root mean square fluctuations (RMSF) of the protein backbone atoms and side chains of residues, respectively (Fig. 8A, B). The averaged RMSD values are 0.25 nm, 0.23 nm, 0.26 nm, and 0.26 nm for the TSA, Hit 1, Hit 2, and Hit 3, respectively. RMSD for all the simulated complexes was below 0.3 nm which indicated the overall stability of hit compounds in the catalytic pocket of HDAC3 (Fig. 8A).

**Figure 9:**
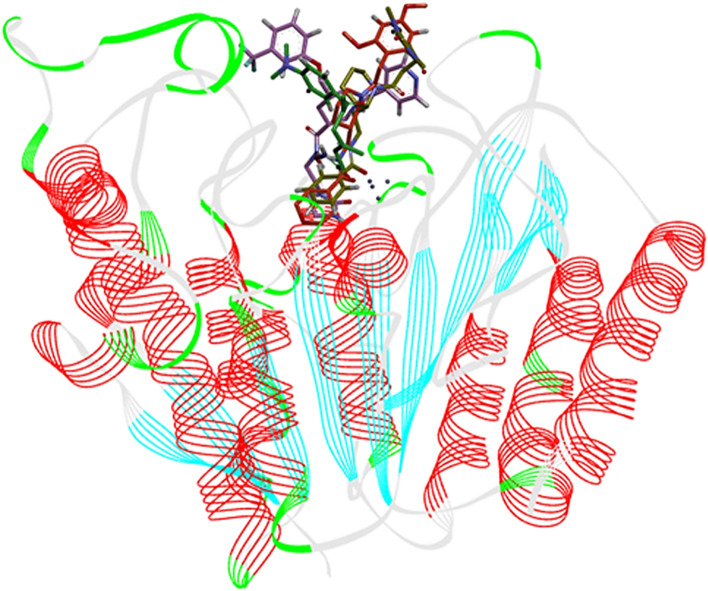
The binding patterns of the reference inhibitor (TSA) and three hit compounds in the active site pocket of HDAC3. The figure is drawn using DS v3.5.

The Hit 1 showed the lowest RMSD as compared to reference inhibitor (TSA), Hit 2, and Hit 3 compounds throughout the 50 ns of simulations. The calculated RMSF values for all the simulated complexes were below 0.3 nm which signifies the good stability for simulated complexes of hit compounds and HDAC3 during simulation. The catalytic residues involving in hydrogen bonding and hydrophobic interactions with TSA and hit compounds showed the least fluctuations throughout 50 ns of simulations which is evident by the < 0.1 nm RMSF value of respective residues (S2 Table). The large fluctuations (0.2 nm to 0.54 nm) were noticed for the loop regions of HDAC3 during the simulations (S2 Table). Further, the binding modes of TSA and three hit compounds were analyzed by superimposing the representative structures which showed a similar binding pattern in the catalytic pocket of HDAC3 where they coordinated with the Zn^2+^ (Fig. 9). Furthermore, the compactness of HDAC3 in presence of hit compounds were assessed by calculating the radius of gyration (Rg) (Fig. 8C). The Rg predicts the compactness of the protein owing to the spatial arrangement of secondary structures. The Rg values (1.92 nm to 2.0 nm) of HDAC3 represent the compactness of HDAC3 during simulations due to the stable behavior of secondary structures.

**Figure 10:**
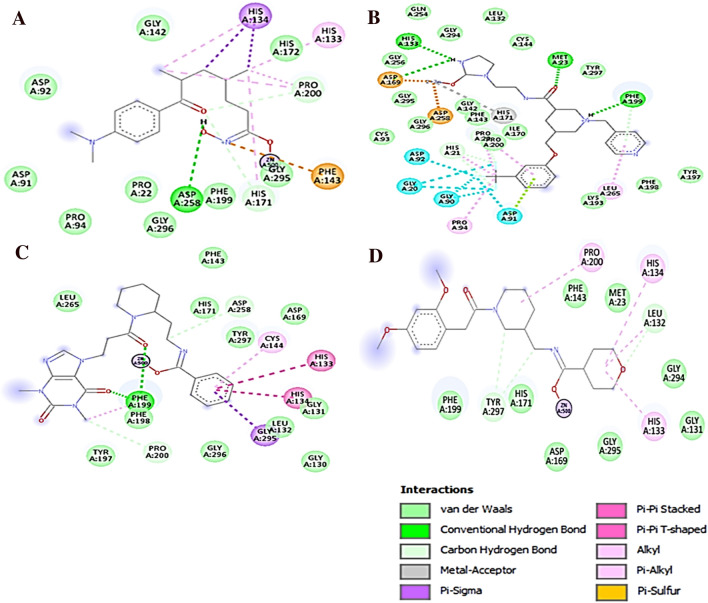
Intermolecular hydrogen bond, hydrophobic and Zinc co-ordinated interactions between the simulated complexes of HDAC3 and Hit compounds; (**A**) Reference (TSA), (**B**) Hit 1, (**C**) Hit 2 and (**D**) Hit 3. The figure is drawn using DS v3.5.

The intermolecular hydrogen bonding, π-stacking, and hydrophobic interactions between the simulated complexes of HDAC3 with TSA and hit compounds are depicted in Figs. 8D, 10 and Table 5. TSA was involved in hydrogen bonding interaction with catalytic Asp258 residue of HDAC3 by maintaining a 2.94 Å distance (Fig. 10A, Table 5).

**Table 5:** Molecular interactions between simulated complexes of TSA and screened Hit compounds with HDAC3 (4A69.PDB).

Molecule name	Atoms involved in H-bonds	Distance 1–2 in (Å)	Angle (°)	Hydrophobic and Van der Walls contacts	Fig. Ref
TSA-4A69	TSA-O21-H…OD2-Asp258	2.94	127.87	His133, His134, Phe143, His171, Pro200, Gly142, Pro22, Asp91, Asp92, Pro94, His172, Phe199, Gly295, Gly296	10a
	TSA-C-H…O-Asp91	2.41	143.49		
	TSA-O11…H-C-His171	2.84	124.60		
	TSA-O11…H-C-Pro200	2.11	165.06		
	TSA-O22…H-C-Gly295	2.31	139.66		
	TSA-O22…Zn^2+^	2.07	–		
Hit 1-4A69	Hit 1-O33…H-N-Met23	2.80	91.06	His21, Pro22, Pro94, Leu265, Cys93, Leu132, Gly142,Cys144, Phe143, Ile170, Lys193, Tyr197, Phe198, Gln254, Gly256, Asp258, Gly295, Gly294, Gly296, Tyr297, Phe199	10b
	Hit 1-N–H…NE2-His133	2.29	123.18		
	Hit 1-N…O-Gly142	3.39	102.35		
	Hit 1-N–H…OD1-Asp169	2.71	125.26		
	Hit 1-N–H…O-Phe199	2.58	156.93		
	Hit 1-F…H-C-Pro22	2.65	118.53		
	Hit 1-F…H-C-His21	2.41	149.55		
	Hit 1-F…H-C-Pro94	2.33	128.57		
	Hit 1-F…H-C-Asp92	2.91	109.52		
	Hit 1-O5…Zn^2+^	2.18	–		
Hit 2-4A69	Hit 2-N–H…NE2-His134	2.66	153.60	π-Stacked_His133, π-Stacked_His134, Leu132, Cys144, Phe199, Leu265, Gly295, Met23, Asp92, Gly131, His133, His134, Gly142, Phe143, Tyr197, Phe198, Gln254, Gly294, Tyr297	10c
	Hit 2-O33…H-N-Phe199	1.94	121.45		
	Hit 2-O33…H-N-Phe199	2.00	146.15		
	Hit 2-O33…H-C-His171	3.09	111.28		
	Hit 2-O33…H-C-Phe198	2.77	122.40		
	Hit 2-O34…H-C-Phe198	2.24	130.34		
	Hit 2-C-H…O-Pro200	2.87	116.38		
	Hit 2-O5…Zn^2+^	2.00	–		
Hit 3-4A69	Hit 3-N–H…NE-His134	3.07	151.45	Leu132, His133, His134, Phe143, His171, Phe199, Pro200, Met23, Asp92, Gly131, Gly142, Cys144, Asn196, Phe198, Gln254, Leu265, Gly294, Gly295	10d
	Hit 3-C-H…O-Gly131	2.35	91.82		
	Hit 3-C-H…O-Tyr297	2.21	113.41		
	Hit 3-O25…Zn^2+^	1.86	–		

The non-classical CH…O hydrogen bonding interactions were also analyzed for simulated complexes due to their importance in the stabilization of protein–ligand complexes^57^. TSA participated in CH…O type of hydrogen bonding interactions with Asp91, His171, Pro200, and Gly295 residues of HDAC3 (Table 5).

TSA was coordinated with Zn^2+^ by 2.07 Å distance. The Hit 1 was involved in hydrogen bonding interactions with Met23, His133, Gly142, and Phe199 from the active site pocket of HDAC3 (Fig. 10B). Hit 1 was coordinated with catalytic Zn^2+^ by maintaining a 2.18 Å distance. The Hit 1 also formed hydrophobic and van der walls interactions with catalytic residues of HDAC3 that could provide structural stability to Hit 1 in the catalytic pocket of HDAC3 (Table 5). The Hit 1 was involved in π-alkyl interactions with Pro22 and Leu265, and π-lone pair interactions with Asp91. The fluorine mediated weak interactions between Hit 1 with Gly20, Gly90, Asp91 and Asp92 were observed in the simulated complex.

**Figure 11:**
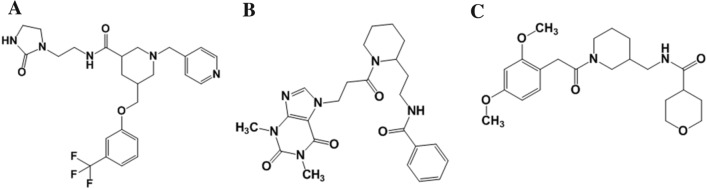
The 2-D structures of screened hit compounds. The figure is drawn using ChemDraw Ultra v12.0.2.

Further, the Hit 2 and HDAC3 complex was found stable during the simulation (Fig. 8). In this complex, the catalytic Phe199 residue participated in the bifurcated interaction with Hit 2 (Fig. 10C). Hit 2 was involved in the weak CH…O interactions with His171, Phe198, and Pro200 catalytic pocket residues of HDAC3 (Table 5). Hit 2 was coordinated with Zn^2+^ by retaining 2.0 Å distance. The benzene ring of Hit 2 participated in two π-stacking interactions with His133 and His134 residues. The Leu132, Cys144, Phe199, Leu265, and Gly295 residues were contributed in hydrophobic interactions with Hit 2 (Fig. 10C). The Hit 3 participated in weak interactions with Gly131 and Tyr297 residues and coordinated with Zn^2+^ from the catalytic pocket of HDAC3 (Fig. 10D and Table 5). Hit 3 was involved in π-alkyl interactions with His133, His134 and Pro200 residues. The residues Leu132, His133, His134, Phe143, His171, Phe199, and Pro200 contributed to hydrophobic interactions with Hit 3. The hydrogen bonding, π-interactions, and hydrophobic contacts from three hit compounds are comparable to the TSA. Therefore, these hit compounds may act as potent and selective inhibitors of HDAC3 and may be used for the treatment of cancer subtypes. The 2D structures of final hit compounds are given in Fig. 11.

### Binding free energy calculation by using the MM-PBSA method

The binding affinity of TSA, Hit 1, Hit 2, and Hit 3 toward the HDAC3 were investigated by calculating the MM-PBSA (Table 6). Total binding energy (ΔG_binding_) of TSA, Hit 1, Hit 2, and Hit 3 in complex with HDAC3 was found as − 61.08 ± 29.11, − 62.39 ± 27.34, − 150.78 ± 17.81, − 39.17 ± 77.79 kJ/mol respectively. This suggested that Hit 1 and Hit 2 have a strong binding affinity toward HDAC3 as compared to the reference inhibitor TSA. The VDW energy (ΔE_vdw_) has a major contribution to the total binding energy and is more favorable to complex formation with all three hit compounds as compared to TSA.

**Table 6:** The binding free energy (kJ/mol) between the simulated complexes of TSA and screened hit compounds with HDAC3 was calculated by the MM-PBSA method.

Complex	∆E_vdw_	∆E_elec_	∆G_polar_	∆G_non-polar_	∆G_binding_
TSA	− 148.01 ± 10.23	− 24.49 ± 5.22	125.06 ± 39.31	− 13.64 ± 0.43	− 61.08 ± 29.11
Hit 1	− 301.51 ± 22.80	8.41 ± 11.07	257.39 ± 16.95	− 26.68 ± 1.39	− 62.39 ± 27.34
Hit 2	− 257.69 ± 6.5	16.77 ± 10.25	111.72 ± 15.37	− 21.59 ± 1.20	− 150.78 ± 17.81
Hit 3	− 226.86 ± 8.15	27.68 ± 6.01	178.69 ± 71.64	− 18.69 ± 0.82	− 39.17 ± 77.79

**∆**E_vdw_, ∆E_ele_, ∆G_polar_, ∆G_non-polar_ and ∆G_binding_ represented van der Waals energy, electrostatic energy, polar solvation energy, nonpolar solvation energy and binding energy, respectively.

Similarly, the ∆G_non-polar_ energy was lowest for all three hit compounds as compared to TSA. This signifies that the ΔE_vdw_ and ∆G_non-polar_ energies participated in the favorable binding of all hit compounds to HDAC3. However, the ∆E_elec_ energy of TSA was lowest as compared to all three hit compounds. The estimated total binding energy suggested that the screened hit (Hit 1 and Hit 2) compounds have a better binding profile as compared to reference TSA and therefore these hits can be acts as a good inhibitor of HDAC3.

**Figure 12:**
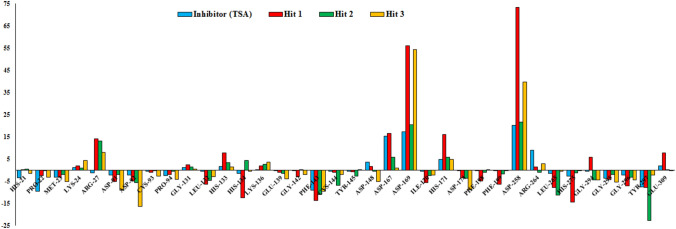
Energetic contribution of individual residues from simulated protein–ligand complexes of Inhibitor (TSA, Blue color), Hit 1 (Red color), Hit 2 (Green color) and Hit 3 (Yellow color) with HDAC3. The figure is drawn using Microsoft Excel 2013 v15.

To investigate the residual contribution of each residue of HDAC3 in complex formation with TSA and all three hits, we estimated the residual binding free energy decomposition using the MM-PBSA (Fig. 12). The Pro22, Asp91, Asp92, Pro94, His134, Phe143, Phe199, Gly295 and Gly296 residues of HDAC3 were favorably contributed in binding with TSA (Figs. 10, 12). The Met23, Asp91, Asp92, Cys93, Pro94, Gly142, Phe143, Leu170, Phe198, Phe199, Leu265, Gly295, Gly296 and Tyr297 were involved in binding with Hit 1.

Further, the Leu132, Cys144, Phe198, Phe199, Leu265, Gly295, Gly296 and Tyr297 participated in favorable binding with Hit 2. Similarly, the Met23, Leu132, His134, Phe143, Phe199, Gly294, Gly295 and Tyr297 were involved in binding with Hit 3. The Met23 and Phe199 were involved in hydrogen bonding interactions with Hit 1 and Hit 2. The His134 was π-stacked with Hit 2. The Leu132, His134, Cys144, Leu265 and Gly295 were involved in π-alkyl interactions with Hit 1, Hit 2 and Hit 3. The Pro22, Asp91, Asp92, Pro94, Gly142, Phe143, Leu170, Phe198, Gly294, Gly295 and Gly296 were involved in van der Waals interactions with TSA and all three hits (Fig. 10). The van der Waals energy was contributed more to the total binding energy of all complexes which was supported by the large number of residual interactions observed in simulated structures (Fig. 10).

### Principle component analysis (PCA)

**Figure 13:**
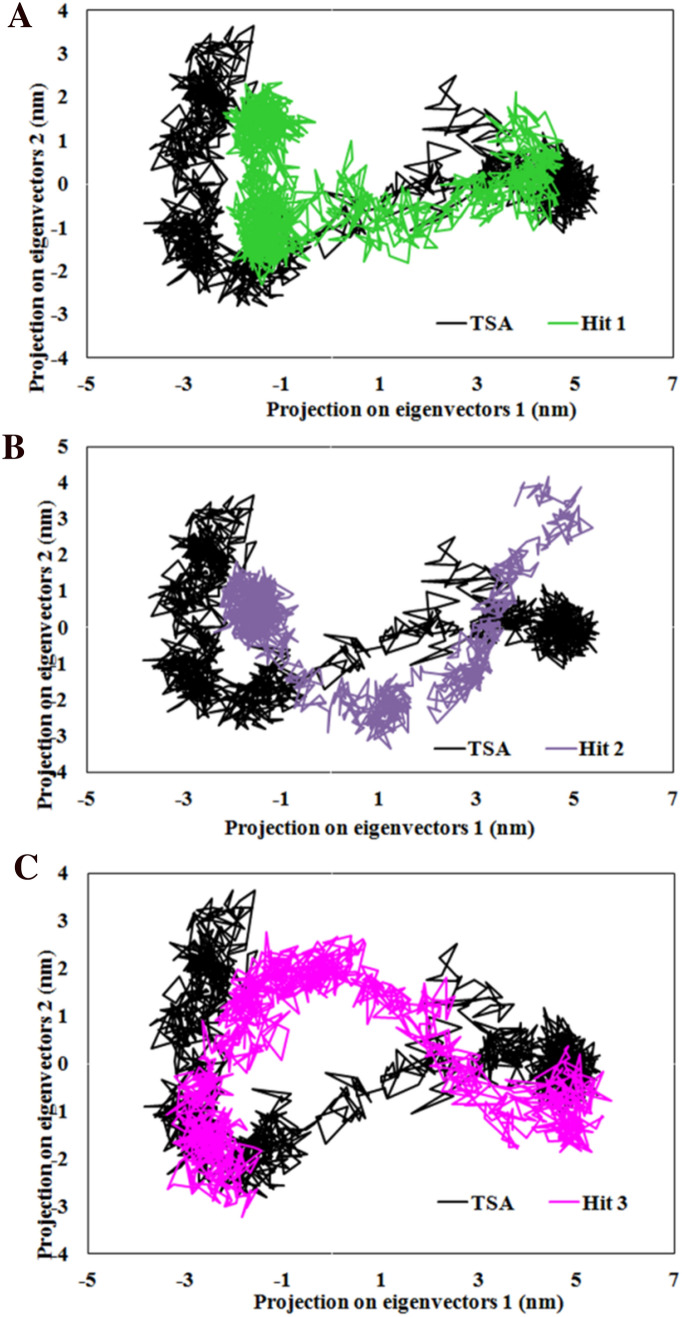
PCA plot showing most significant principal components of motion of the Cα atoms of HDAC3 in complex with (**A**) Hit 1/TSA, (**B**) Hit 2/TSA, and (**C**) Hit 3/TSA. The figure is drawn using Microsoft Excel 2013 v15.

To assess the impact of binding of hit compounds on the overall collective motions of HDAC3 in complex with TSA and hit compounds, we have performed PCA using simulated trajectories (Fig. 13). The covariance matrix was constructed for all simulated complexes which predicted the trace values for complexes of HDAC3 with TSA, Hit 1, Hit 2 and Hit 3 are 22.24 nm^2^, 14.42 nm^2^, 16.17 nm^2^ and 21.63 nm^2^, respectively. These trace values indicated that the Hit 1, Hit 2, and Hit 3 showed less atomic flexibility in complex with HDAC3 as compared to the HDAC3-TSA complex. Interestingly, Hit 1 exhibited the lowest amplitude of fluctuations at the atomic level when compared to Hit 2 and Hit 3. Further, the eigenvectors and their respective eigenvalues were calculated by using the diagonalized covariance matrix to investigate the projection of eigenvectors in the conformational space with their amplitude. The first two eigenvectors (1 and 2) were used to evaluate the concentrated motions of HDAC3 upon the binding of hit compounds. The 2D plots of projections of the first two vectors showed that the predicted clusters of all hit compounds have an extended nature in conformational space with PC 1, as compared to PC 2 (Fig. 13). The binding of Hit 1 to HDAC3 produced favorable conformational changes with the formation of confined clusters as compared to TSA (Fig. 13A). However, the perturbed direction of motions of HDAC3 was observed in Hit 2 and Hit 3 (Fig. 13B, C). Furthermore, the magnitude of eigenvector (0.01153 nm) was found lowest for the Hit 1-HDAC3 complex as compared to TSA (0.01779 nm), Hit 2 (0.01730 nm), and Hit 3 (0.012 nm). This indicated that the binding of Hit 1 to HDAC3 was more favorable than TSA and other hits.

### Protein–ligand interaction profile (PLIP) analysis of training set (FDA approved drugs) and final simulated hit compounds

The results of PLIP analysis of training set compounds and simulated hits showed a concrete relationship between the salient chemical features (Hypo 1) present in training set compounds (FDA approved drugs) and molecular interactions observed in simulated hits in complex with HDAC3. The structural information for five drug molecules such as TSA (5EEF, 16), LBH (5EF8, 1), JNJ-26481585 (6HSK, 2), ACY-1215 (5WGL, 1), and SHH (4LXZ, 11) was retrieved from the crystal structures whereas, for NVP-LAQ824, MS-275 and VPA drugs were obtained from the earlier docking studies^58–60^. Total 31 co-crystal structures of these five drugs were downloaded from the protein data bank and analyzed for protein–ligand interactions profiles using PLIP online server and DS. Out of these, one representative interaction profile of each drug is compared to the interaction profiles of simulated hits (Tables 5 and 7). These seven drugs coordinated to the catalytic Zn^2+^ in all crystal structures and docking studies performed against HDACs. These drugs were involved in hydrogen bonding interactions with His, Lys, Gly, Tyr, Ser, Glu, and Asp residues from the active site pocket of HDACs. The TSA, LBH, JNJ-26481585, NVP-LAQ824, and MS-275 were involved in π-stacking (Phenylalanine and Histidine) and hydrophobic interactions with catalytic residues of HDACs (Table 7).

**Table 7:** The combined protein–ligand interactions profiles of training set compounds obtained from the PLIP server and DS.

PDB ID	π-Stacking, van der Walls and Hydrophobic Interactions	Metal interactions	Hydrogen bond
**Trichostatin (TSA)**			
HDAC6 (5EEF)	π-Stacking_Phe202, His82, Pro83, Ser150, Gly201, Phe202, His232, Gu360, Trp261, Gly361, Asp323 and Gly362	**TSA**, Asp323, Asp230 and His232	TSA-O….His193 TSA-O…Lys330 TSA-O….Gly361 TSA-O….Tyr363
**Panobinostat (LBH)**			
HDAC6 (5EF8)	π-Stacking_Phe583, π-Stacking_Phe643, Asp460, His463, His573, His574, Gly582, His614, Asp705, Leu712 and Gly743	**LBH**, Asp612, His614 and Asp705	LBH-O…Tyr745 LBH-O…Ser531
**Quisinostat (JNJ-26481585)**			
HDAC8 (6HSK)	π-Stacking_Phe152, π-Stacking_His180, π-Stacking_Phe208, Asp101, His142, His143, Gly151, Gly206, Phe207, Pro209, Gly210, Leu274 and Gly304	**JNJ-26481585**, Asp178, His180 and Asp267	JNJ-26481585-O…Tyr306
**Dacinostat (NVP-LAQ824)**			
HDAC1 (Docking)	π-Stacking_His178, π-Stacking_Phe150 π-Stacking_His205, π-Stacking_His28	**NVP-LAQ824**, His141 and Asp176	LAQ824-N…Glu98, LAQ824-N…Gly149, LAQ824-O…Asp99
**Recolinostat (ACY-1215)**			
HDAC6 (5WGL)	His462, Pro464, Ser531, Gly582, Phe643, Phe583, His614, Asp705, Pro711, Leu712 and Gly743	**ACY-1215**, Asp612 and His614, Asp705	ACY-1215-O…Tyr745, ACY-1215-N…His573, ACY-1215-N…His574
**Vorinostat (SAHA)**			
HDAC-2 (4LXZ)	His33, Pro34, Gly154, Phe155, His145, His183, Phe210, Leu276 and Gly306,	**SHH**, Asp181, His183 and Asp269	SHH-N…Asp104 SHH-N…His146 SHH-O…Tyr308
**Entinostat (MS-275)**			
HDAC2 (Docking)	π-Stacking_Pro34, Glu67, Thr70, Lys71, Lys149, Leu166, Ala199, Lys284, Asp345, Lys347, His349, Ile351 and Ser351,		MS-275…Asn331
**Valproic acid (VPA)**			
HDAC2 (Docking)	Met35, Leu144, Phe155, Cys156, Phe210 and Leu276	**VPA**, Asp181, His183 and Asp269	VPA-O…Tyr308

On the other hand, the active compounds including TSA, CUDC-907, LBH, JNJ-26481585, NVP-LAQ824, and ACY-1215 from the training set were mapped with all chemical features of Hypo 1. The moderately active SHH and MS-275 missed one HYP chemical feature whereas, the least active VPA missed one RA and one HYP chemical feature when aligned to Hypo 1.

The highly active compounds contained all chemical features while moderately active and least active compounds missed one or more chemical features when aligned to Hypo 1. This inferred that Hypo 1 was able to discriminate the active, moderately active, and least active compounds more efficiently. The RA chemical feature present in Hypo 1 was governed to π-interactions present in the co-crystallized active (TSA, LBH, JNJ-26481585, and NVP-LAQ824) and docked (MS-275) drug molecules with HDACs (Table 7). The HBA chemical feature facilitated the coordinated interaction with Zn^2+^ and hydrogen bonding interaction of drugs with catalytic residues of HDACs, except MS-275. Further, the two HYP features of Hypo 1 represented the hydrophobic nature of drugs which is evident by the observed hydrophobic interactions between the drugs and active site pocket residues of HDACs (Table 7). Interestingly, the analyzed interactions data of training set compounds and its correlation with chemical features present in Hypo 1 suggested that HBA, RA, and HYP are very essential features needed to equip molecules as potent inhibitors of HDACs. The hydrophobic interactions are playing a crucial role in adopting proper conformation of drugs in the catalytic pocket of HDACs to inhibit its catalytic activity. The absence of HYP and RA features leads to reduced activity of drug molecules against HDACs. This analysis exclusively evidenced the good quality of Hypo 1 and delves to establish the vital link between the chemical features present in approved drugs and their ability to form molecular interactions with HDACs.

Similarly, the analyzed PLIP profiles of simulated structures of hit compounds showed a close resemblance in the presence of chemical features and their molecular interactions with HDAC3 (Fig. 7, Table S4). All three hits were significantly coordinated with the Zn^2+^ and involved in hydrogen bonding interactions with catalytic residues of HDAC3 (Table 5). This is justified by the mapped HBA feature by hit compounds when aligned to Hypo 1 (Fig. 7). The RA feature is representing the π-type of interactions that were noticed in all simulated complexes of hits and HDAC3. Further, the observed hydrophobic interactions in simulated complexes of hits and HDAC3 were supported by the two HYP features mapped with Hypo 1. The PLIP analysis enabled us to establish a good correlation between the presence of chemical features in the training set and hit compounds and their molecular interactions with HDAC3. Moreover, the performed ligand-based pharmacophore modeling, virtual screening, and molecular dynamics simulation studies may pave the way to understand the molecular mechanism of HDAC3 inhibition by screened hit compounds. Therefore, the screened hits may act as potent and selective inhibitors against HDAC3 and may be used for the treatment of different types of cancers.

## Discussion

The epigenetic modifications are mainly involved in the transcriptional repression of tumor suppressor genes and activation of oncogenes^61^. HDAC3 is an epigenetic regulator that deacetylates the lysine residues from the histone proteins which resulted in chromatin condensation. The elevated expression of HDAC3 has been associated with the progression of various cancer subtypes, neurodegenerative disorders, and many other human diseases. Therefore, it is considered as a potential therapeutic target for designing new potent inhibitors to treat human diseases^5–7,10,26,27,34–37,51^. The computationally advanced techniques and comprehensive knowledge of pharmacophoric features of HDAC3 inhibitors may help to design more potent and selective inhibitors^51–53^. Therefore, we performed the ligand-based pharmacophore modeling of HDAC3 inhibitors to predict the salient pharmacophore features that estimate the inhibitory activity scale of new lead candidates. Further, the homogeneity of the biological assay is one of the important aspects of the 3D-QSAR study, therefore training and test dataset compounds were collected from the same biological assay.

The statistical parameters such as cost value, correlation coefficient, fit value, and RMSD were used to generate the pharmacophore model. The best pharmacophore hypothesis 'Hypo 1' contains one HBA, one RA, and two HYP chemical features which represented the high correlation coefficient (*R*^2^ = 0.994). The correlation coefficient values for ten generated models were greater than 0.93 and the highest correlation of Hypo 1 demonstrated the strong predictive ability (Table 1). This is evident by the lowest RMSD (0.373) value that indicates the minor difference between the estimated and experimental activities of HDAC3 inhibitors (Table 2). Further, the first nine hypotheses contain similar chemical features that might be the reason for the lower RSMD (< 1.0) value while the replacement of HBA by the HBD feature in the last hypothesis resulted in the higher RMSD (1.19) value. In addition, the presence of the HBD feature in Hypo 10 was attributed to low-cost difference, small correlation coefficient as well as fit value. Total cost values of 10 generated pharmacophore models ranged from 102.51 to 115.96. Hypo 1 has scored the total cost value close to the fixed cost (98.65) value whereas, the total cost value of Hypo 10 was far away from the fixed cost as compared to other generated models. The activity values of 23 out of 24 training set compounds were estimated within their experimental activity range while one active compound 9 was estimated as moderately active (Table 2). The calculated error values for the experimental and estimated activities are within one order of magnitude (< 2.4) that justifies the good predictability of Hypo 1. The chemical features of Hypo 1 were present in all active compounds of the training set whereas, two or three chemical features were present in moderately active and inactive compounds. This anticipated that Hypo 1 can discriminate the training set compounds based on their activity scales.

Further, the earlier studies performed using the 3D-QSAR, ligand, and structure-based pharmacophore modeling showed similar chemical features, as observed in Hypo 1 (Table 1)^45–50^. Interestingly, the HBA, RA, and 2HYP chemical features of Hypo 1 were found similar as described in the earlier ligand-based pharmacophore modeling of HDAC1 inhibitors. Pharmacophore modeling of HDAC2 and HDAC6 consisted of HBA, RA, and HYP features, in addition to HBD^45,48^. The HBD feature observed in our last hypothesis was previously reported in structure-based pharmacophore modeling of HDAC6 inhibitors^47^. The RA feature was absent in pharmacophore models of HDAC8 and HDACs inhibitors whereas, the HYP feature was absent in the 3D-QSAR model of HDAC1^44,49^. The correlation coefficient of the predicted Hypo 1 was higher than the other pharmacophore models of HDACs (Table 1-SI). This again inferred that the quality of the Hypo 1 model was good and could efficiently estimate the activity of HDAC3 inhibitors.

Furthermore, the randomly generated 19 spreadsheets in the Fischer randomization run did not show better statistical parameters over the Hypo 1, this thereby confirmed that Hypo 1 was not generated by chance. In addition, the validation of Hypo 1 by the external test set overestimated three compounds while all remaining 57 compounds were correctly estimated to their respective experimental activity scales (Table 3). A high correlation coefficient between the experimental and estimated activities of training and test set compounds indicates the good predictive ability of Hypo 1. The screening of ~ 1.2 million compounds followed by subsequent drug-like properties filtration resulted in 174 hits. The 22 final hits were retrieved after the docking study which helps to narrow down the false-positive rate. Moreover, three-hit compounds that mapped all the chemical features of Hypo 1 and resulted as best hits in docking studies were subjected to 50 ns of MD simulations. Simulations have helped to assess the binding mode and time-dependent behavior of hits in the active site pocket of HDAC3. The TSA and all three hits were bound in the active site pocket of HDAC3 (Fig. 9). The RMSD, RMSF and Rg values were below 3 nm which presented the good stability of hits in the catalytic pocket of HDAC3 during simulations. All hits were strongly coordinated with Zn^2+^ and facilitated the molecular interactions such as hydrogen bonds, hydrophobic and π-interactions with active site residues of HDAC3. HDACs are the Zn^2+^ dependent enzymes therefore, the Zn^2+^ coordination by hits may lead to inhibition of HDAC3 activity. This was supported by the strong molecular interactions between hits and HDAC3 observed during MD simulations. The estimated total binding energy of Hit 1 and Hit 2 were more favorable for complex formation with HDAC3 as compared to TSA and Hit 3. Also, the calculated residue-wise decomposition of the binding energy of HDAC3 has supported the chemical features present in Hypo 1 and molecular interaction patterns observed in simulation studies. Additionally, the PCA analysis was found to be in tune with docking, MD simulations and MM-PBSA results.

Moreover, the analyses of the protein–ligand interactions showed that all co-crystallized and docked drugs were coordinated with Zn^2+^ and involved in hydrogen bonding, hydrophobic and π-interactions with the active site residues of various HDACs isomers (Table 7). Similar interactions were observed in simulated hit compounds which are supported by the pharmacophore features present in Hypo 1 (Fig. 2, Table 5). Remarkably, the interaction profiles suggested that HBA, RA, and HYP are very essential chemical features required for hit compounds to acts as potent inhibitors against HDAC3 and other isoforms. The PLIP analysis established a significant correlation between the chemical features of Hypo 1 and molecular interactions from the training set and simulated hit compounds. The pharmacophore modeling and simulations may pave the way to elucidate the essential chemical features needed for the potent activity of HDAC3 inhibitors towards catalytic inhibition of HDAC3. The screened hit compounds may act as selective inhibitors of HDAC3 and may be used for the treatment of cancer subtypes.

## Conclusion

We have developed a ligand-based pharmacophore model ‘Hypo 1’ which consisted of one HBA, one RA, and two HYP salient chemical features with an excellent correlation coefficient value of 0.994. Fischer's randomization analysis inferred that Hypo 1 is the statistically best-fit hypothesis, and not generated by chance. The validation of Hypo 1 using an external test set yielded a good correlation coefficient between the predicted and experimental IC_50_ values. This delineated the good predictive ability of Hypo 1. Database screening followed by drug-likeness analysis identified hits that can act as potent HDAC3 inhibitors. These hits were further narrowed down to 22 by molecular docking using the Goldscore and Chemscore as a cut-off of reference inhibitor (TSA). The three hit compounds and TSA showed comparable results in MD simulations by retaining Zn^2+^ coordination, π-interactions, hydrogen bonding, and hydrophobic interactions. These results are comparable with earlier crystallographic data of FDA approved drugs which co-crystallized with HDACs. Further, the PLIP analysis showed a close resemblance in molecular interactions of the training set and hit compounds with the presence of chemical features in Hypo 1. Therefore, the screened hit compounds may act as a potent and selective inhibitor of HDAC3 and may find applications for the treatment of various cancer subtypes and other human disorders regulated by HDAC3.

## Methods

### Compounds selection and preparation of the dataset

The selection of a training set of compounds is a crucial step for the generation of a pharmacophore model which subsequently determines the quality of the generated pharmacophores. The dataset of 84 known HDAC3 inhibitors with diverse structural scaffold and different inhibitory activities were obtained from earlier literature reports^62–66^. The selected compounds were divided into the training set and the test set. The training set compounds were used to build the 3D-QSAR pharmacophore model and the generated models were validated using the test set compounds. The 24 HDAC3 inhibitors were used as a training set compounds for the generation of the 3D-QSAR pharmacophore model; while the generated pharmacophore models were validated using a test set of 60 compounds (S1 Table). Both dataset compounds possessed chemical as well as structural diversity with a different range of HDAC3 inhibitory concentrations (IC_50_ values) and spanned over the four orders of magnitude. The IC_50_ values of the training set compounds ranged from 0.84 nmol/L to 260,000 nmol/L. The training set compounds were categorized as active (IC_50_ < 100 nmol/L, +++), moderately active (100 nmol/L ≤ IC_50_ < 10,000 nmol/L, ++) and inactive (IC_50_ ≥ 10 000 nmol/L, +) based on their IC_50_ values. Similarly, the test set compounds were classified based on their IC_50_ values. The 2D chemical structures of 84 compounds were drawn using ChemDraw Ultra v12.0.2 software (https://chemistrydocs.com/chemdraw-ultra-12-0/) and their 3D structures were generated using Discovery Studio v3.5 (DS) software (https://discover.3ds.com).

### Pharmacophore model generation

Before generating the pharmacophore model, *the Feature Mapping Protocol* was employed to identify the salient chemical features of the training set compounds that facilitate the selective inhibition of HDAC3. The HDAC3 inhibitors possessed hydrogen bond acceptors (HBA), hydrogen bond donors (HBD), ring aromatic (RA) and hydrophobic regions (HYP) are the mapped features. The pharmacophore models were generated using these features from the *3D-QSAR Pharmacophore Generation Protocol* available in DS by correlating the known experimental activity (IC_50_) values of inhibitors with their chemical structures. The *BEST algorithm* was used to generate the low-energy conformations of the compounds. The energy threshold value was set to 20 kcal/mol^67^. The uncertainty value was set to 3 while other parameters were kept at their default values. The top ten quantitative hypotheses were generated based on the activity values offered by the training dataset compounds by using the Debnath method^68^. Debnath method suggests that if the model has a high correlation coefficient (*R*^2^), the lowest total cost, the lowest root mean square deviation (RMSD), maximum fit values, and the total cost close to the fixed cost and far from the null cost, such models can be considered as the best quantitative hypothesis. The reliability of the hypothesis depends on the difference between the total cost of the generated hypothesis and the null hypothesis.

### Pharmacophore model validation

The best pharmacophore model should qualify the desired statistical values, predict the accurate activity of the compounds, and should have the ability to retrieve active compounds from the chemical databases. The best hypothesis was subjected to validations by Fisher's randomization and the external test set method. The statistical significance of the best-selected model was computed by employing Fischer’s randomization method^69^. This method checks the correlation between the chemical structure and the biological activity of a compound. This method also overrules the possibility of chance correlation for the pharmacophore model development and ensures that the model was not generated by chance. The 95% of confidence level was set during the 3D-QSAR pharmacophore generation process, which generated nineteen random spreadsheets^70^. This was done by randomizing the activity of these compounds by using the same features and parameters that were used to generate the original pharmacophore hypothesis. During this process, if any of the random pharmacophore hypotheses showed better statistical values than the best hypothesis (Hypo 1), then it was considered that the Hypo1 was generated by random correlation^71^. Further, the external test set of 60 chemically diverse compounds was used to determine the ability of the Hypo1 that could predict and classify the compounds to their correct experimental activity scale other than training set compounds. The predicted and experimental activity values were plotted to observe the range of correlation between them. Low energy conformations of test set compounds were generated using the same protocols used for the training set compounds. The *BEST* algorithm with the *Flexible* fitting option from the *Ligand Pharmacophore Mapping* module was used during the test set validation.

### Virtual screening and prediction of drug-likeness

Virtual screening of chemical databases was conducted to identify novel compounds with new scaffolds that can trigger or inhibit the activity of the HDAC3. The pharmacophore-based (or ligand-based) virtual screening of the diverse chemical databases can be used as a resource for the identifications of novel and potential candidate leads in the drug discovery process. The best pharmacophore model delineates the chemical functionalities responsible for the bioactivities of potential drugs, thereby evident its use in performing a database search. The validated best pharmacophore model was selected as a 3D query to screen the NCI, Asinex, Chembridge, and Maybridge chemical databases which contain millions of compounds with diverse structural scaffolds. The *Fast* and *Flexible* options from the *Ligand Pharmacophore Mapping* protocol of DS were used for the database screenings. The compounds from screened databases that fit all the features of the best pharmacophore model were retrieved as hit compounds. Further, the drug-like physicochemical properties of hit compounds were predicted by using Lipinski's rule of five^72^. Lipinski’s rule of five estimates the absorption and intestinal permeability of drugs. According to this rule, the well-absorbed drugs should possess less than 10 hydrogen bond acceptor groups, less than 5 hydrogen bond donor groups, a molecular weight of less than 500 Da, a Log *P* value of less than 5, and rotatable bonds less than 10. Furthermore, the absorption, distribution, metabolism, excretion, and toxicity (ADMET) properties of each compound were calculated using the *ADMET Descriptors* protocol in DS. This protocol investigated the ability of compounds to cross the blood–brain barrier (BBB), optimal solubility, good intestinal absorption, non-inhibition to CYP2D6, and non-hepatotoxicity^67^. The compounds scoring better-estimated activities and fulfilled all drug-likeness properties were selected for molecular docking studies.

### Molecular docking of hit compounds with HDAC3

The molecular docking of screened hit compounds with the target protein has emerged as a very effective tool in the modern drug development process^38,39,50,51^. Docking can be used to find out the most appropriate conformation and binding modes of hit compounds, interactions of small molecules with catalytic site residues of target proteins, and also estimate their binding affinities^73–76^. The training set compounds and all hit compounds were docked in the binding site of HDAC3. The docking studies were performed using the Genetic Optimization for Ligand Docking (GOLD) program v5.2.2 (https://www.ch.cam.ac.uk/computing/software/gold-suite). ^77^ The genetic algorithm from GOLD allows partial flexibility to protein and full flexibility to ligands during the docking process. The crystal structure of HDAC3 (PDB code: 4A69) was downloaded from the protein data bank (PDB, rcsb.org) and used for the molecule docking^54^. As a part of preparing a target protein, the water molecules were removed from the protein and hydrogen atoms were added to calculate the bond orders for protein and ligand molecules. The orientations of all histidine tautomers from the catalytic pocket of HDAC were transformed into the ND1H protonation states as observed in the crystal structure^54^. All the atoms within 10 Å of the co-crystallized ligand in the crystal structure of HDAC3 were defined as the binding pocket of the protein. The binding affinity of the ligand to the protein was predicted using default scoring function such as Goldscore and rescoring was done using Chemscore. The 100 docking poses were generated for each ligand and the best poses were selected based on high Goldscore and low Chemscore. Furthermore, the docked poses were validated by analyzing the molecular interactions such as hydrogen bonds, hydrophobic interaction, and metal interactions between the ligand and the active site residues from the catalytic pocket of HDAC3. The final docked complexes of hit compounds with HDAC3 were subjected to molecular dynamics (MD) simulation studies.

### Molecular dynamics (MD) simulation

The MD simulations of 50 ns were performed on stable docked complexes of HDAC3 with the top three screened hit compounds and most active compound from the training set using the GROMACS 2018 package (https://www.gromacs.org) with a Gromos96 force field^78,79^. The PRODRG, an online webserver was used to generate the topology files for ligands^80^. The system was solvated with a TIP3P water model around the HDAC3 with 10 Å from the edged cubic box and neutralized by Na^+^ counter ions. Initially, the system was energy minimized by 10,000 steps using the steepest descent method to remove the possible unfavorable contacts from initial structures until the tolerance of 2000 kJ/mol was achieved. The energy minimized system was then subjected to equilibration in three different steps. A constant temperature controlled by a Nose–Hoover thermostat was applied for 100 ps at 300 k in the first phase of equilibration^81^. Later, a 100 ps NPT ensemble was applied at 1 bar of pressure followed by 50 ns of the production run under the same ensembles. In the simulation, the pressure of the system was maintained using the Parrinello-Rahman barostat method^82^. The protein backbone of HDAC3 was restrained and solvent molecules with counter ions were allowed to move during the equilibration process. All bonds to a hydrogen atom were restrained by applying the LINCS algorithm using a 2 fs of time step^83^. The particle mesh ewald method was employed to calculate the long-range electrostatic interactions^84^. The Coulombic and van der Waals interactions were calculated by employing the cut-off distance of 9 Å and 10 Å, respectively. The MD simulations were performed by releasing all constraints along with the periodic boundary conditions to avoid edge effects^85^. The 2 fs of time step was used throughout the simulation and the coordinate data of trajectories were stored at every picosecond (ps). The simulation results were analyzed using GROMACS, VMD (https://www.ks.uiuc.edu/vmd/), and DS.

### Binding-free energy calculation by MM-PBSA

The binding free energy of protein–ligand complexes of all three hits and reference TSA with HDAC3 were calculated using the Molecular mechanics Poisson-Boltzmann surface area (MM-PBSA) method^86^. The average binding energy was calculated by analyzing 20 snapshot structures of each simulated complex from the last 20 ns of MD trajectories. The g_mmpbsa tool of GROMACS was employed to calculate the contribution of different energetic parameters such as van der Waals (ΔE_vdw_), electrostatic (ΔE_elec_), non-polar solvation (ΔG_nps_), and polar solvation (ΔG_Ps_) energy in the total binding energy. Also, the residual contribution of key residues in binding free energy was calculated by the MmPbSaDecomp.py python script.

Binding energy was calculated as,

$$\begin{aligned} & \Delta {\text{G}}_{{{\text{bind}}}} = \Delta {\text{E}}_{{{\text{MM}}}} + \, \Delta {\text{G}}_{{{\text{Solv}}}} \\ & \Delta {\text{E}}_{{{\text{MM}}}} = \, \Delta {\text{E}}_{{{\text{vdw}}}} + \, \Delta {\text{E}}_{{{\text{elec}}}} \\ & \Delta {\text{G}}_{{{\text{Solv}}}} = \, \Delta {\text{G}}_{{{\text{nps}}}} + \, \Delta {\text{G}}_{{{\text{ps}}}} \\ \end{aligned}$$

### Principle component analysis (PCA)

Principle component analysis is an advanced technique in MD simulation to study the conformational dynamics of protein-inhibitor complexes^87,88^. PCA was performed on the conformational ensembles selected from the MD trajectories of simulated complexes of HDAC3 with TSA and screened hit compounds. The PCA was constructed for the displacement of C_α_ atoms of HDAC3 to evaluate its global motion in complex with TSA and hit compounds. PCA was carried out using standard protocol available in the GROMACS Software package^78^. In the first step, the covariance matrix was constructed by using the gmx_covar tool which calculates eigenvectors and their eigenvalues. Further, the projections of the eigenvector along with the first two components were analyzed by the gmx anaeig tool. The first two eigenvectors (PC1 and PC2) having the highest eigenvalues which represent the large-scale concentrated motions were selected to plot the 2D projections of each independent trajectory.

### Protein–ligand interactions profile (PLIP) analysis

The protein–ligand interaction profiles of simulated complexes of HDAC3 with screened hit compounds, and co-crystallized FDA approved and investigational drugs from the training set were analyzed using PLIP online program (https://plip-tool.biotec.tu-dresden.de/plip-web/plip/index) and DS software^89^. The training set contains 9 FDA approved and investigational drugs such as Trichostatin A (TSA), Fimepinostat (CUDC-907), Panobinostat (LBH), Quisinostat (JNJ-26481585), Dacinostat (NVP-LAQ824), Recolinostat (ACY-1215), Vorinostat (SHH), Entinostat (MS-275) and Valproic acid (VPA) which were used for the 3D-QSAR pharmacophore model generation. The intermolecular electrostatic interactions including hydrogen bonds, hydrophobic contacts, and metal interactions were predicted. The PLIP profiles of hit compounds were compared with the approved drugs from the training set.

## Supplementary Information

Supplementary Information.
