# Supplementary material for: Identification of novel leads as potent inhibitors of HDAC3 using ligand-based pharmacophore modeling and MD simulation
Source: Sci Rep. 2022 Feb 2;12:1712. doi: 10.1038/s41598-022-05698-7 (PMC8810932; doi:10.1038/s41598-022-05698-7)
Supplement: Supplementary file 1 — Supplementary Information. [file 41598_2022_5698_MOESM1_ESM.docx]

**Supplementary Information**

**Identification of novel leads as potent inhibitors of HDAC3 using ligand-based pharmacophore modeling and MD simulation**

Navanath Kumbhar,^1^ Snehal Nimal,^1^ Sagar Barale,^2^ Subodh Kamble,^3^ Rohit Bavi,^3^ Kailas Sonawane,^2^ Rajesh Gacche.^1*^

1. Department of Biotechnology, Savitribai Phule Pune University Pune, Maharashtra (MS), 411007, India.

2. Department of Microbiology, Shivaji University, Kolhapur, Maharashtra (MS) 416004, India.

3. Structural Bioinformatics Unit, Department of Biochemistry, Shivaji University, Kolhapur, Maharashtra

(MS) 416004, India.

4. School of Chemical Science, Punyashlok Ahilyadevi Holkar Solapur University, Solapur 413255, India.

**Table S1.** A representation of the total 60 chemically diverse test set compounds used for Hypo 1 validation. The experimental IC_50_ values (nmol/L) are shown in parentheses for each compound.

| 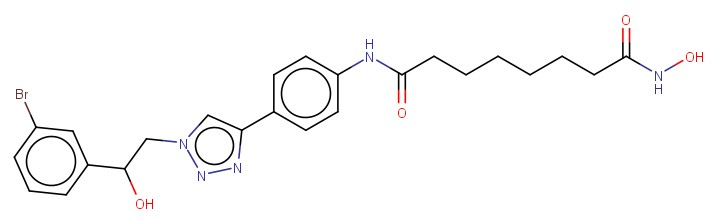 | 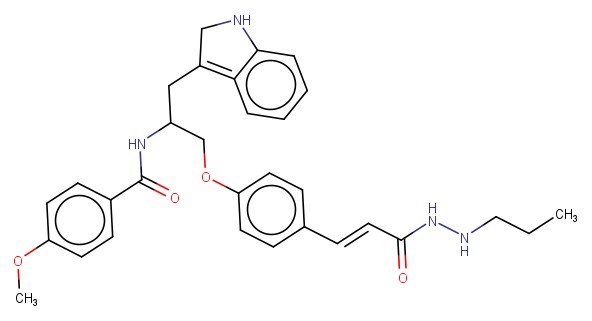 | 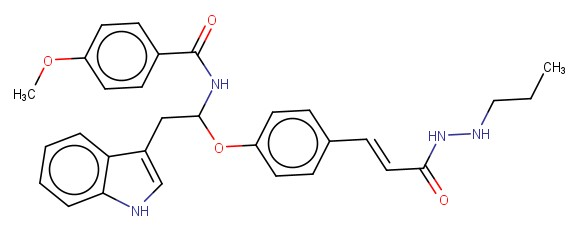 |
| --- | --- | --- |
| Compound 1 (2.7) | Compound 2 (2.8) | Compound 3 (2.8) |
| 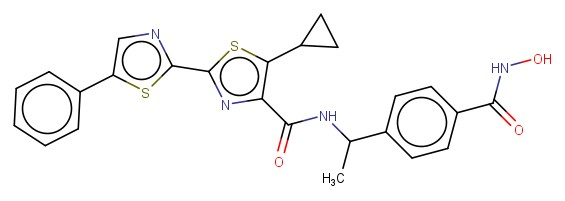 | 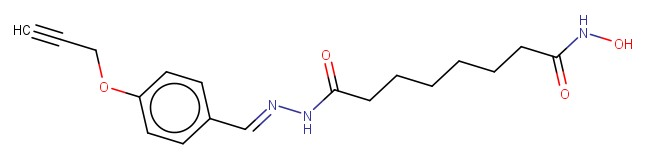 | 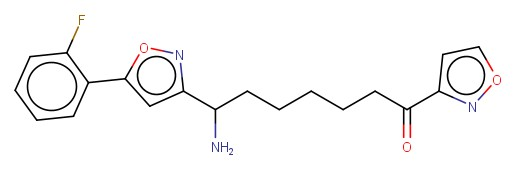 |
| Compound 4 (3.0) | Compound 5 (3.1) | Compound 6 (3.2) |
| 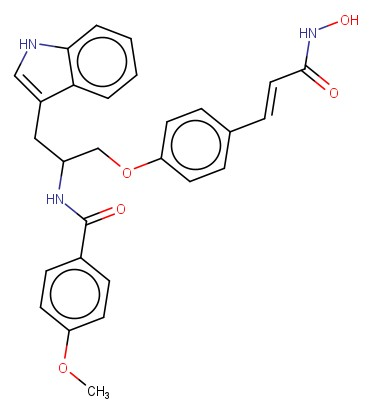 | 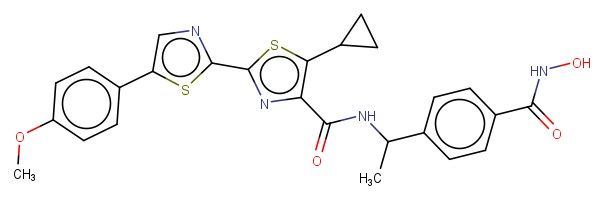 | 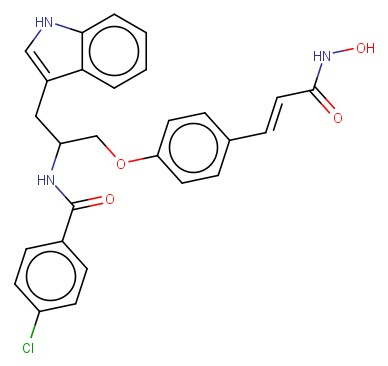 |
| Compound 7 (3.2) | Compound 8 (3.8) | Compound 9 (3.9) |
| 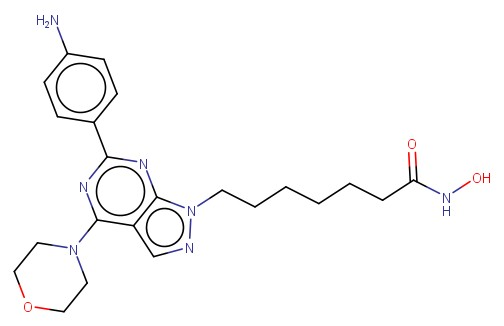 | 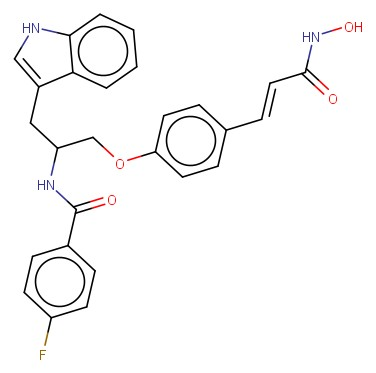 | 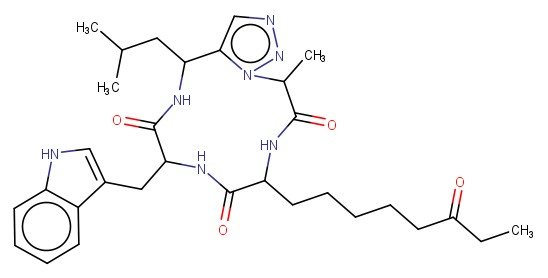 |
| Compound 10 (4.2) | Compound 11 (5.5) | Compound 12 (9) |
| 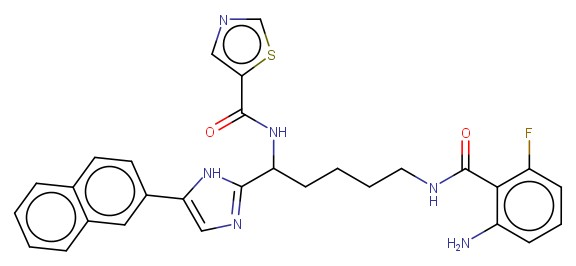 | 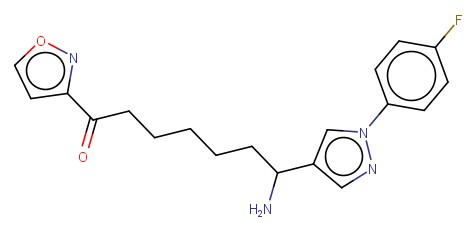 | 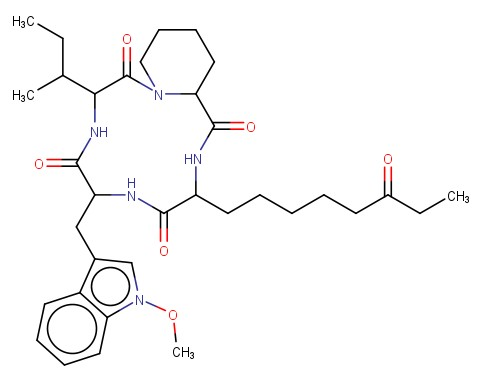 |
| Compound 13 (9.3) | Compound 14 (13) | Compound 15 (13) |
| 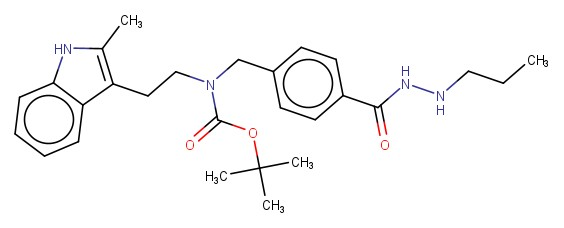 | 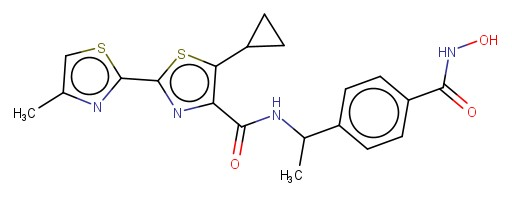 | 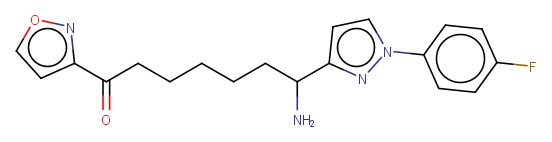 |
| Compound 16 (13) | Compound 17 (13.9) | Compound 18 (15) |
| 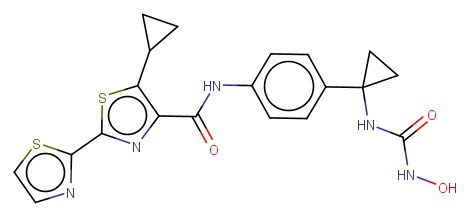 | 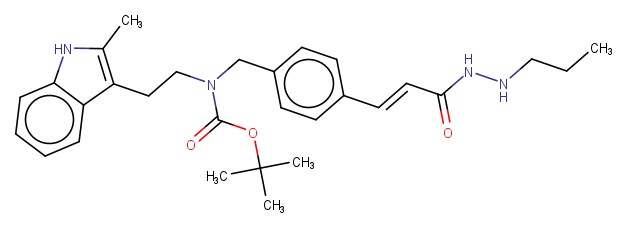 | 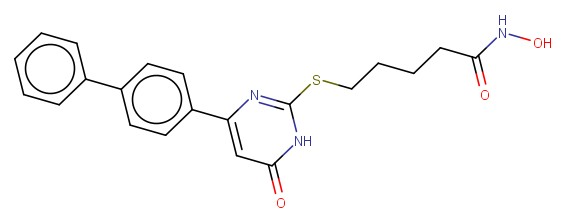 |
| Compound 19 (16.3) | Compound 20 (17) | Compound 21 (20) |
| 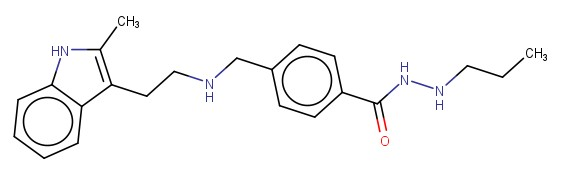 | 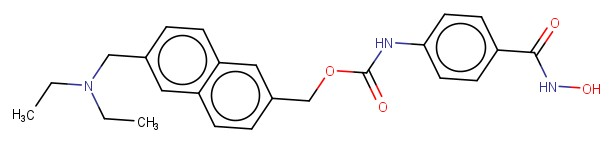 | 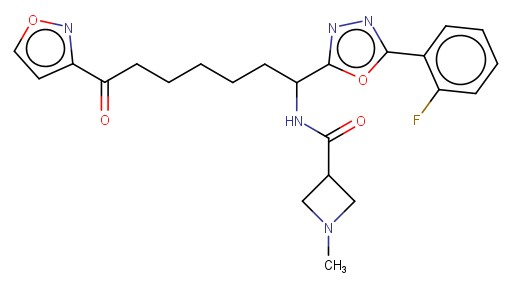 |
| Compound 22 (20) | Compound 23 (21) | Compound 24 (23) |
| 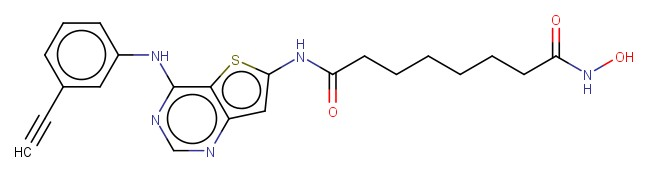 | 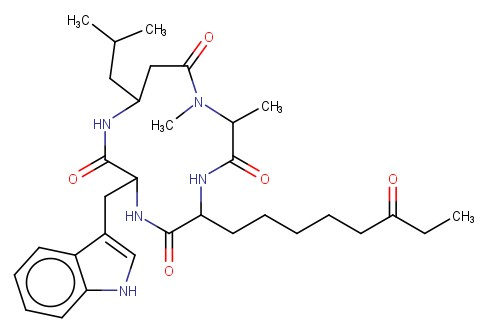 | 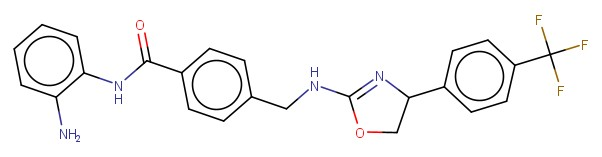 |
| Compound 25 (24.7) | Compound 26 (30) | Compound 27 (34) |
| 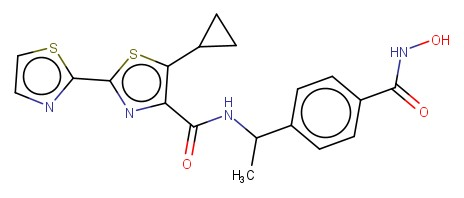 | 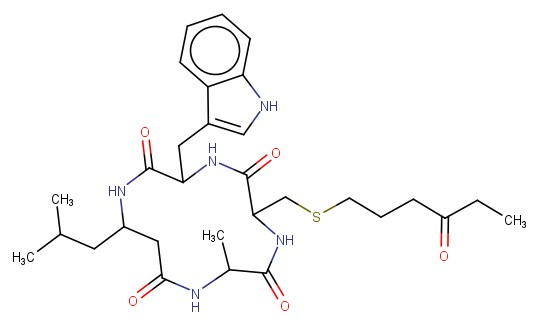 | 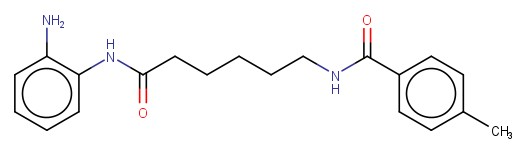 |
| Compound 28 (42.5) | Compound 29 (62) | Compound 30 (63) |
| 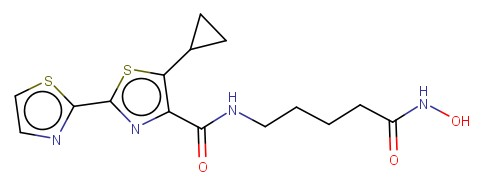 | 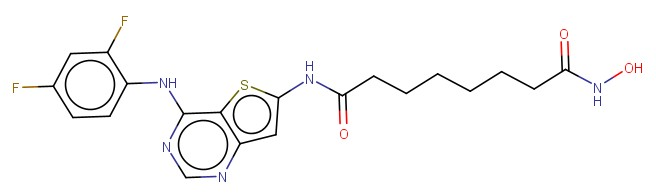 | 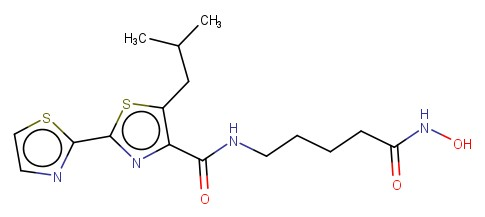 |
| Compound 31 (74) | Compound 32 (100) | Compound 33 (100) |
| 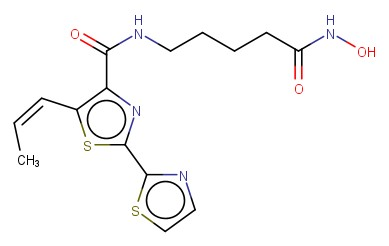 | 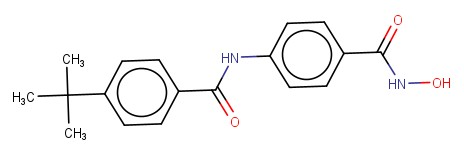 | 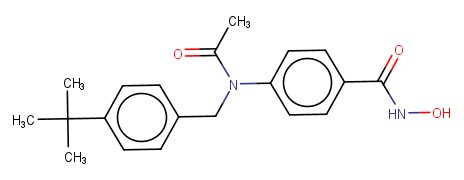 |
| Compound 34 (110) | Compound 35 (187) | Compound 36 (276) |
| 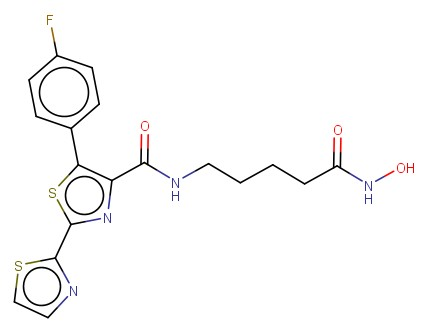 | 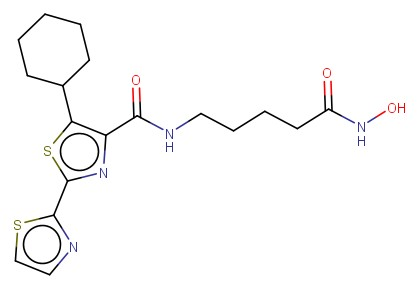 | 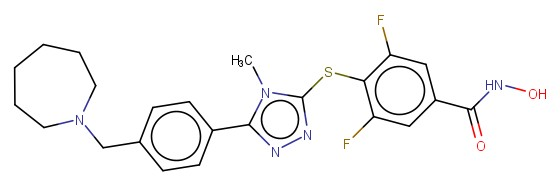 |
| Compound 37 (310) | Compound 38 (330) | Compound 39 (354) |
| 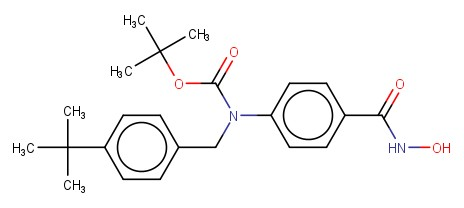 | 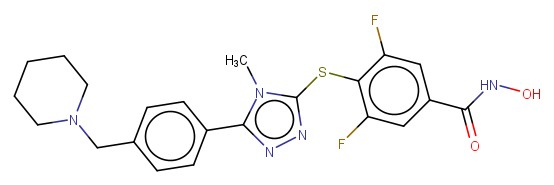 | 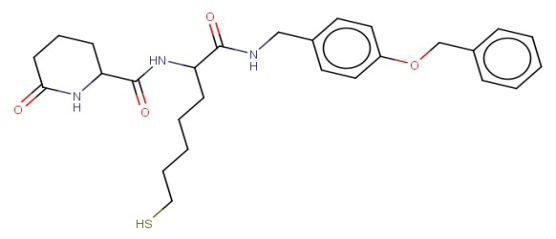 |
| Compound 40 (374) | Compound 41 (464) | Compound 42 (495) |
| 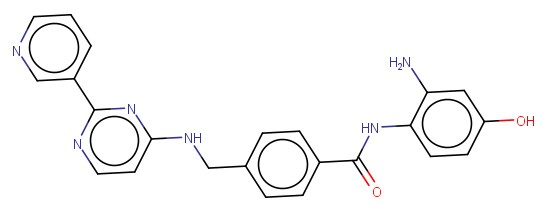 | 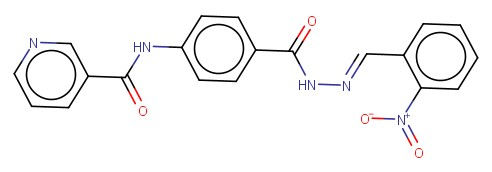 | 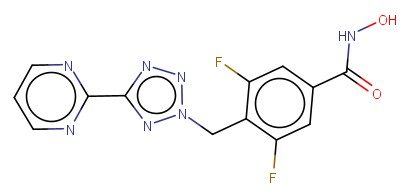 |
| Compound 43 (719) | Compound 44 (887) | Compound 45 (967) |
| 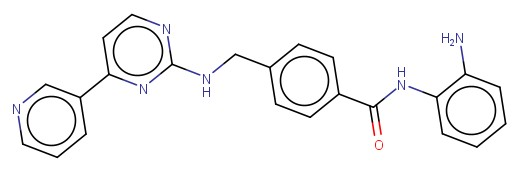 | 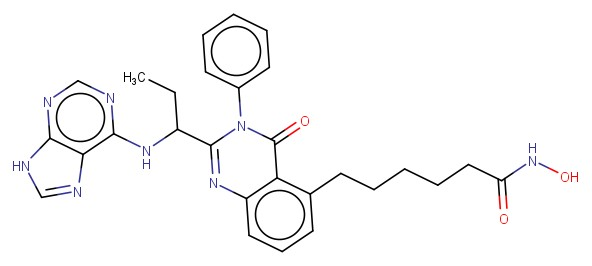 | 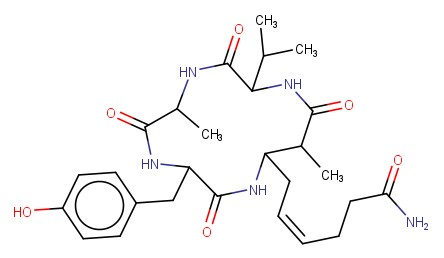 |
| Compound 46 (1700) | Compound 47 (2400) | Compound 48 (3000) |
| 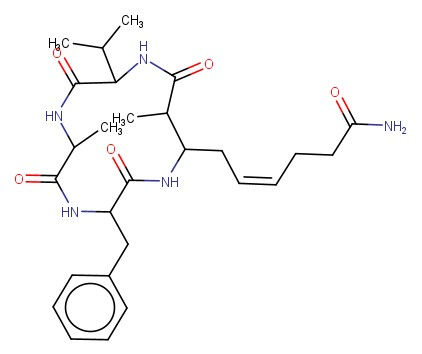 | 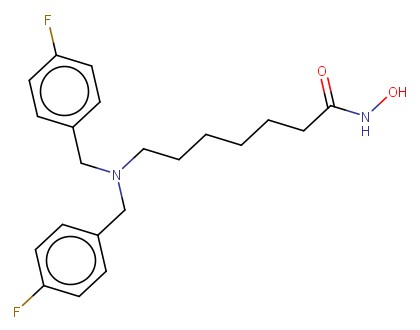 | 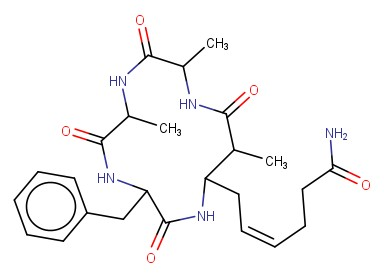 |
| Compound 49 (3200) | Compound 50 (3600) | Compound 51 (3700) |
| 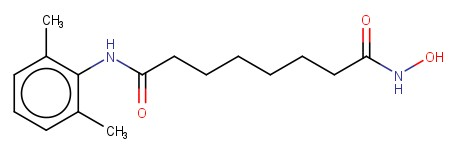 | 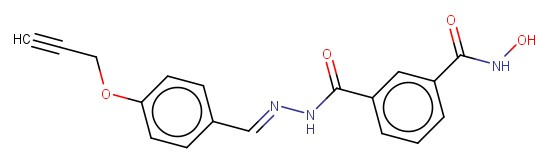 | 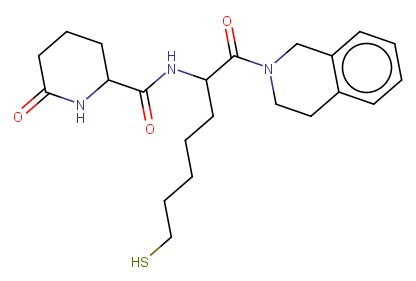 |
| Compound 52 (4989) | Compound 53 (6200) | Compound 54 (6330) |
| 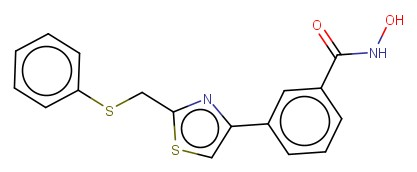 | 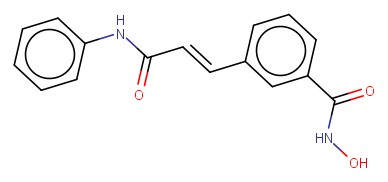 | 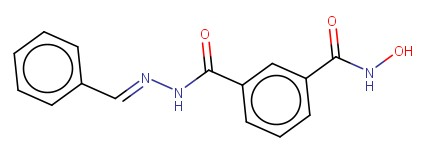 |
| Compound 55 (12000) | Compound 56 (18000) | Compound 57 (18000) |
| 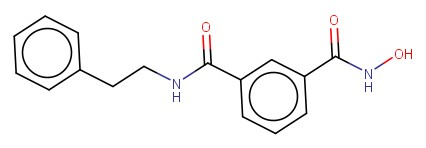 | 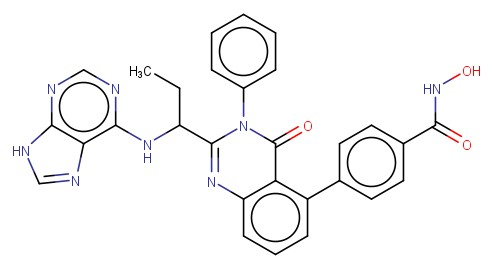 | 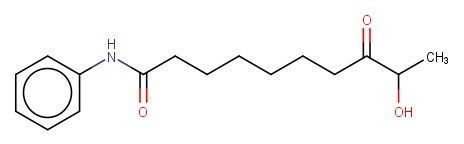 |
| Compound 58 (23000) | Compound 59 (48000) | Compound 60 (50200) |

Note: These figures are drawn using ChemAxonMarvin suite software (https://chemaxon.com/products/marvin)


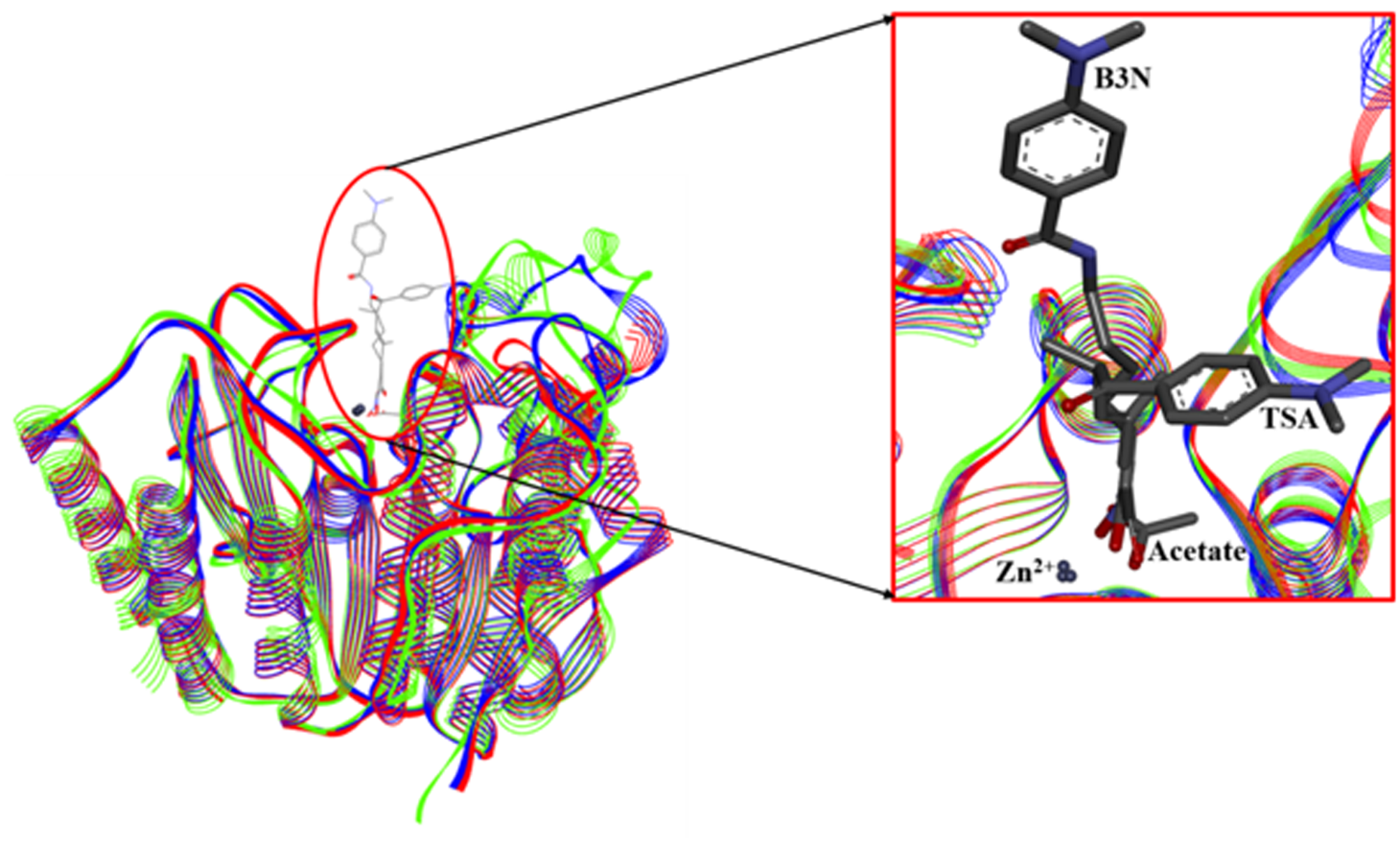


**Figure S1.** Active site comparison between HDAC3 (4A69.PDB, Acetate ion) and HDAC8 (3EW8.PDB, B3N and 1T64.PDB, TSA). The figure is drawn using DS v3.5.

**Table S2.** Calcualted RMSF for catalytic residues from HDAC3 for 50 ns of MD simulations.

| **Simulated complex** | **HDAC3 Residues** | **RMSF (nm)** |
| --- | --- | --- |
| TSA-4A69 | His133  His134  Phe143  His171  Pro200  Asp258  Gly295 | 0.06  0.06  0.10  0.10  0.26  0.07  0.06 |
| Hit 1 | His21  Pro22  Met23  Asp92  Pro94  His133  Gly142  Phe199  Leu265 | 0.10  0.08  0.08  0.10  0.08  0.04  0.05  0.08  0.12 |
| Hit 2 | His133  His134  Cys144  His171  Phe198  Phe199  Pro200  Leu265  Gly295 | 0.07  0.07  0.13  0.05  0.12  0.10  0.10  0.11  0.10 |
| Hit 3 | Gly131  Leu132  His133  His134  Phe143  His171  Ph199  Pro200  Tyr297 | 0.05  0.05  0.06  0.07  0.08  0.08  0.25  0.27  0.07 |
| Loop region of HDAC3 | 9-21  70-87  180-185  195-219  259-267  320-345 | 0.12-0.23  0.13-0.54  0.11-0.24  0.11-0.39  0.11-0.26  0.13-0.32 |

**Table S3.** Predicted pharmacophore features of HDACs inhibitors with differnet methods.

| **Name of HDACs** | **Features** | **Correlation coefficient** | **Method** | **Reference** |
| --- | --- | --- | --- | --- |
| HDAC1 | HBA, HBD, RA | *R^2^_training_* = 0.958  *R^2^_test_* = 0.794 | 3D-QSAR modeling | 1 |
| HDAC1 | HBA, RA, 2HYP | *R^2^_training_* = 0.946 | Ligand-based pharmacophore model | 2 |
| HDAC2 | HBA, HBD, RA, HYP | *R^2^_training_* = 0.759 | Ligand-based pharmacophore model | 3 |
| HDAC6 | HBD, RA, 2HYP | - | Structure based pharmacophore modeling | 4 |
| HDAC6 | HBA, HBD, RA, HYP | *R^2^_training_* = 0.980  *R^2^_test_* = 0.940 | Ligand-based pharmacophore model | 5 |
| HDAC8 | HBA, 2HBD, HYP | *R^2^_training_* = 0.948  *R^2^_test_* = 0.879 | Ligand-based pharmacophore model | 6 |
| HDAC | HBA, HBA, HBD, HYP | *R^2^_training_* = 0.891  *R^2^_test_* = 0.853 | Ligand based Pharmacophore modeling | 7 |

**Table S4.** The protein-ligand interactions profiles of simulated Hit compounds obtained from PLIP server and DS.

| **PDB ID** | **π-Stacking, van der Walls and Hydrophobic Interactions** | **Metal interactions** | **Hydrogen bond** |
| --- | --- | --- | --- |
| TSA | Pro22, Asp91, Asp92, Pro94, His134, Gly142, Phe143, His171, His172, Phe199, Pro200, Gly295, Gly296 | **TSA**, Asp169, His171, Asp258, Gly295 | TSA-O….Asp258 |
| Hit-1 | His21, Pro22, Cys93, Leu132, Gly142,Cys144, Phe143, Ile170, Lys193, Tyr197, Phe198, Gln254, Gly256, Asp258, Leu265, Gly295, Gly294, Gly296, Tyr297, Phe199 | **Hit-1**, Asp169, His171, Asp258,  Gly256, | Hit-1-O…Met23  Hit-1-N…His133  Hit-1-N…Gly142,  Hit-1-N…Asp169  Hit-1-N…Phe199 |
| Hit-2 | π-stacking_His133, π-stacking_His134, Gly130, Gly131, Leu132, Phe143, Asp169, His171, Tyr197, Phe198, Phe199, Pro200,  Leu265, Gly296, Tyr297 | **Hit-2**, Asp169, His171, Asp258, Gly295 | Hit-2-N…His134  Hit-2-O…Phe199 |
| Hit-3 | Met23, Gly131, Leu132, His134, Phe143, Asp169, His171, Phe199, Pro200, Gly294, Gly295, Tyr297, | **Hit-3**, Asp169, His171, Asp258 | Hit-3-N…His134 |

**References**

1. Sirous, H. et al. Computer-Driven Development of an in Silico Tool for Finding Selective Histone Deacetylase 1 Inhibitors. *Molecules* **25,** 1952 (2020).
2. Liqin,Y. U. Fei, L. I. U. Yadong, C. & Qidong, Y. Pharmacophore Identification of Hydroxamate HDAC 1 Inhibitors. *Chin J Chem* **27**, 557-564 (2009).
3. Kandakatla, N. & Ramakrishnan, G. Ligand Based Pharmacophore Modeling and Virtual Screening Studies to Design Novel HDAC2 Inhibitors. *Adv Bioinform.* **ID 812148,** 11 (2014).
4. Wang, Y. et al. Hierarchical virtual screening of the dual MMP-2/HDAC-6 inhibitors from natural products based on pharmacophore models and molecular docking. *J Biomol Struct Dyn.* **37(3)**, 649-670 (2019).
5. Zeb, A. et al. Investigation of non-hydroxamate scaffolds against HDAC6 inhibition: A pharmacophore modeling, molecular docking, and molecular dynamics simulation approach. *J Bioinform Comput Biol.* **16(3)**, 1840015 (2018).
6. Thangapandian, S. et al. Docking-enabled pharmacophore model for histone deacetylase 8 inhibitors and its application in anti-cancer drug discovery. *J Mol Graph Model* **29(6)**, 894 (2011).
7. Liu, J. et al. Combined pharmacophore modeling, 3D-QSAR and docking studies to identify novel HDAC inhibitors using drug repurposing. *J Biomol Struct Dyn.* **38(2)**, 533-547 (2020).
